# Single-molecule methylation profiles of cell-free DNA in cancer with nanopore sequencing

**DOI:** 10.1186/s13073-023-01178-3

**Published:** 2023-05-03

**Authors:** Billy T. Lau, Alison Almeda, Marie Schauer, Madeline McNamara, Xiangqi Bai, Qingxi Meng, Mira Partha, Susan M. Grimes, HoJoon Lee, Gregory M. Heestand, Hanlee P. Ji

**Affiliations:** 1 Division of Oncology, Department of Medicine, Stanford School of Medicine, Stanford, CA USA; 2 Department of Electrical Engineering, Stanford University, Stanford, CA USA

**Keywords:** Methylation, Single-molecule sequencing, Nanopore, Cell-free DNA

## Abstract

Epigenetic characterization of cell-free DNA (cfDNA) is an emerging approach for detecting and characterizing diseases such as cancer. We developed a strategy using nanopore-based single-molecule sequencing to measure cfDNA methylomes. This approach generated up to 200 million reads for a single cfDNA sample from cancer patients, an order of magnitude improvement over existing nanopore sequencing methods. We developed a single-molecule classifier to determine whether individual reads originated from a tumor or immune cells. Leveraging methylomes of matched tumors and immune cells, we characterized cfDNA methylomes of cancer patients for longitudinal monitoring during treatment.

**Supplementary Information:**

The online version contains supplementary material available at 10.1186/s13073-023-01178-3.

## Background

Malignant tumor cells shed their DNA into the bloodstream of cancer patients in the form of cell-free DNA (cfDNA). Sequencing cfDNA can identify cancer-associated biomarkers and is useful for disease monitoring. This approach is commonly referred to as a liquid biopsy [1–3]. Epigenetic modifications of tumor DNA are of particular interest because of their contribution to cancer development and progression [4]. Characterizing cancer-specific methylation changes has proven to be a highly sensitive and specific modality for liquid biopsies [5–7]. For detecting methylation, cfDNA is typically processed with bisulfite or enzymatic conversion of unmodified cytosines into uracils. Short-read sequencing detects the presence of methylated bases. However, this approach introduces biases such as significant GC skews, DNA damage, PCR amplification bias, and alignment artifacts [8, 9]. Compounding these issues, extracted cfDNA from plasma has low yields. Characterizing cfDNA methylomes from patients remains challenging, particularly with conventional sequencing approaches.

**Fig. 1:**
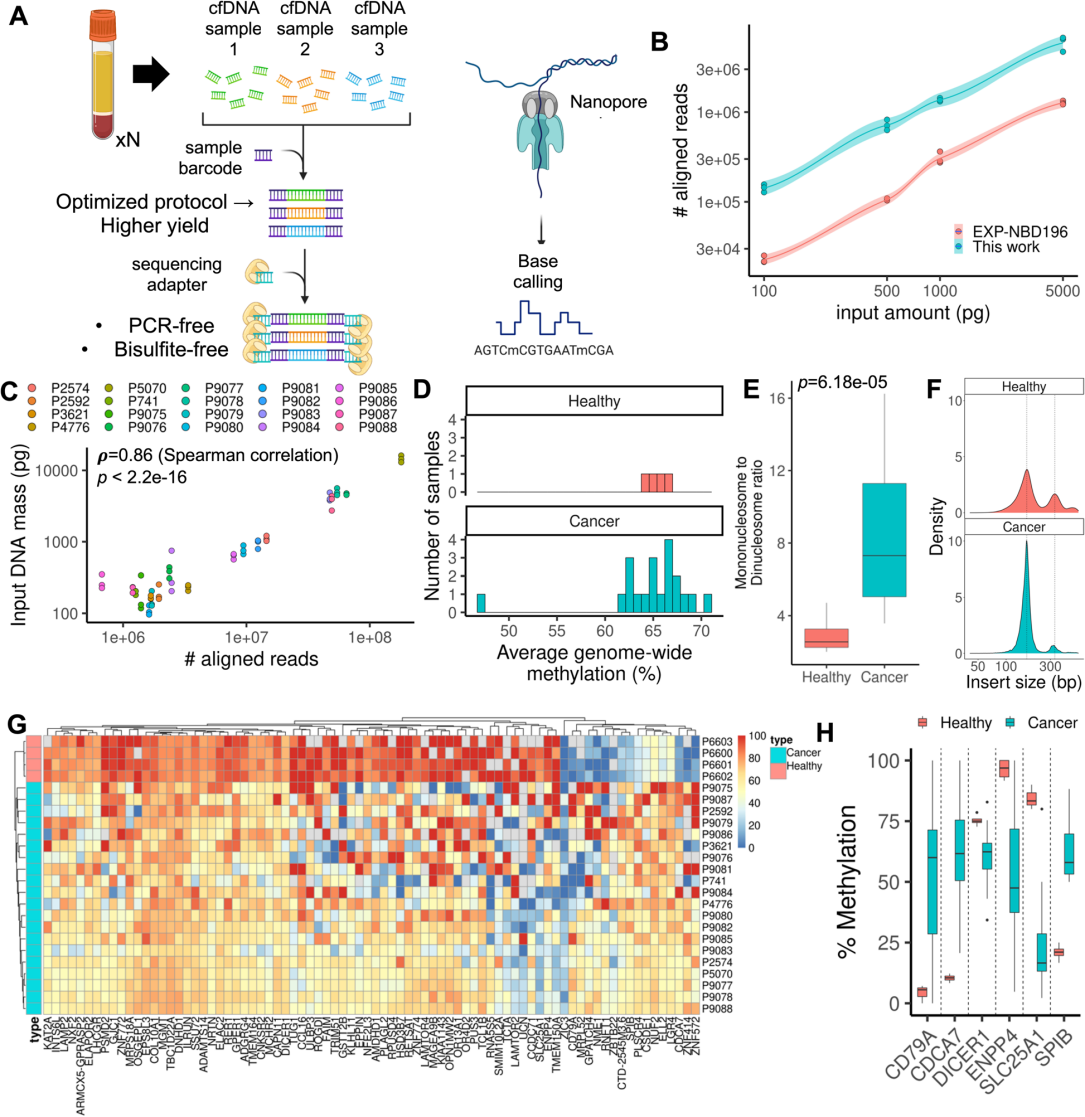
Nanopore sequencing of cfDNA. **A** An optimized protocol for generating cfDNA sequencing libraries enables high-throughput methylation characterization. The key improvement was the optimization of specific end-repair, a-tailing, and ligation conditions to maximize the number of cfDNA fragments available for nanopore sequencing. **B** Cell-free DNA library comparison. An optimized workflow enables approximately an order of magnitude increase in sequencing yield versus the conventional protocol. **C** Sequencing yield correlation with input cfDNA. Fluorometric quantification was performed on cancer patient-derived cfDNA samples and compared to the aligned sequencing yield. Each patient is shown as a separate color in triplicate. Correlation and significance value are annotated on the plot. **D** Genome-wide methylation quantification. The degree of methylation across the genome was computed for healthy and patient-derived cfDNA. **E** Nucleosome enrichment analysis. The ratio of mononucleosomes to di-nucleosomes was quantified for each tissue type, using a cutoff of 250 bp between mono- and di-nucleosomes. **F** Distribution of fragment sizes. Example fragment sizes are shown for healthy and patient-derived cfDNA. Mono- and di-nucleosome size peaks are annotated with dotted lines to be 167 bp and 334 bp. **G** Methylation profiles of healthy- and patient-derived cfDNA. Gene-level methylation values for each sample were determined, and statistically significant ones (*q* < 0.01) are plotted as a heatmap with the gene-level methylation percentage as the intensities. The heatmap was clustered by gene-level methylation. **H** Differential methylation. Statistically significant differences in methylation between sample types are shown for several selected genes

Addressing these challenges, we developed a single-molecule sequencing approach for efficiently characterizing methylation profiles from the cfDNA of cancer patients (Fig. 1A). This PCR-free process generates sequencing libraries from nanogram amounts or less of cfDNA per sample. We leveraged the Oxford Nanopore platform to identify cfDNA methylation without cytosine conversion. The passage of methylated DNA through the nanopore generates a unique electrical signal compared to unmodified DNA; currently, 5-methylcytosine (5mC) CpG methylation is detected with machine learning algorithms at high accuracy [10, 11]. By eliminating PCR, we avoided GC-biased amplification skews from bisulfite conversion and enabled direct single-molecule counting of cfDNA. The nanopore-based methylation profiles thus directly reflect the native single-molecule state of the cfDNA.

## Methods

### Samples

We obtained informed consent from all patients based on a protocol approved by Stanford University’s Institutional Review Board. Our cohort consists of patients with a positive diagnosis of cancer (*N* = 23) and healthy volunteers (*N* = 5). Blood and tissue samples came from the Stanford Cancer Center, the Stanford Tissue Bank, and the Stanford Blood Center. For a subset of patients enrolled at the Stanford Cancer Center (three patients: P6199, P4822, and P6527), we obtained whole blood samples in Streck or EDTA tubes which were later centrifuged into plasma and PBMC fractions. For these patients, matched tumor tissue was also obtained. Plasma from the Stanford Tissue Bank was obtained as single aliquots in 1-ml cryovials. Tumor tissue was archived by flash freezing in liquid nitrogen and stored at − 80 °C. From the Stanford Blood Center, we obtained whole blood from anonymous donors to serve as healthy controls; these samples were centrifuged into plasma and buffy coat fractions. All samples were stored at − 80 °C before processing.

### Nucleosome DNA controls

To generate DNA fragments modeling the qualities of cell-free DNA, we used the EZ Nucleosomal DNA Prep Kit (Zymo Research). This method uses DNase to digest open chromatin positions and yields a fragment pattern characteristic of cell-free DNA instead of random fragmentation. Briefly, nuclei were processed from whole cells by adding a nuclei prep buffer that lyses the cell membrane but leaves the nuclei membrane intact. Enzymatic DNase digestion then fragments DNA at unprotected locations, after which DNA is purified with the kit’s included components. For nucleosomes from cancer lines, we used cells treated with trypsin.

The Stanford Blood Center provided anonymous donor blood as healthy controls. We used peripheral blood mononuclear cells (PBMCs) for nucleosome preparation. Whole blood was diluted with an equal volume of PBS and added to a SepMate PBMC isolation tube (STEMCELL Technologies) containing Ficoll. The tube was spun at 1200* g* for 10 min before decanting into a new tube. Cells were spun again at 400* g* for 5 min and were washed with PBS. Cells were resuspended in freezing medium (90% FBS/10% DMSO). Isolated PBMCs were then used as input for the nucleosome preparation kit. For the experimental admixtures, the PBMC and cell line nucleosomes were diluted to a target concentration (e.g., 1 ng/μl) and mixed to known ratios. Serial dilutions of this mixture are then performed to simulate lower input amounts.

### Sample processing

Extracted DNA was obtained from tissue biopsies using the Maxwell 16 DNA extraction kit (Promega). Briefly, a small tissue fragment was excised from the tissue sample with a scalpel and deposited into the input well of the DNA purification cartridge. The cartridge was placed into the Maxwell 16 instrument (Promega), and the associated protocol was run. For extracting cell-free DNA, plasma was separated from whole blood by centrifugation. The plasma fraction was pipetted into a Maxwell 16 ccfDNA Plasma kit cartridge (Promega) using the standard instrument protocol. The cellular blood portion was extracted using a Maxwell 16 LEV Blood DNA Kit. Yields were measured by Qubit (Thermo Fisher Scientific). Cell-free DNA was quantified using the AccuBlue NextGen DNA Quantification Kit (Biotium).

### Sequencing library preparation

We developed a protocol for generating sequencing libraries that accommodate the low input amounts of cfDNA and maximize sample barcode adapters’ incorporation rate. A full protocol is available online at https://dx.doi.org/10.17504/protocols.io.4r3l27rjxg1y/v1 [12]. Briefly, 25 μl of extracted cfDNA (out of a typical 50 μl extracted volume; thus corresponding to ~ 0.5 ml of plasma) was diluted with 25 μl of water. The sample DNA underwent end-repair and A-tailing with conditions of 20 °C for 30 min and 65 °C for 30 min (Roche KAPA HyperPrep kit). We ligated native barcodes using 5 μl of each barcoded adapter (EXP-NBD196, Oxford Nanopore Technologies) following the standard reaction volumes in the KAPA HyperPrep workflow. We used a thermocycler for the ligation step for 4.5 h incubation at 20 °C before holding at 4 °C overnight to maximize the ligation yield. These steps provided a higher ligation rate of cell-free DNA molecules to a native barcode adapter than the standard protocol’s shorter end-repair/A-tailing and ligation time (10 min per the standard Oxford Nanopore protocol).

After the ligation step, 88 μl of Mag-Bind Total NGS beads (Omega Bio-Tek; an alternative to Ampure XP beads) were added and mixed to each reaction. After incubation for 5 min, the mixtures were pooled together into a 50-μl centrifuge tube. The beads were magnetized and washed with 80% ethanol using a DynaMag separation rack (Thermo Fisher Scientific) before eluting in 600 μl of 10 mM Tris–HCl pH 8.0 buffer. We performed a second bead cleanup step with 900 μl Mag-Bind Total NGS beads (1.5 × ratio) and the same magnetic rack procedure. The elution solution was 50 μl 10 mM Tris–HCl pH 8.0 buffer.

We improved the preparation of multiplex sequencing libraries. For the Oxford Nanopore platform, multiplexing is restricted to the AMII adapter, which has the same motor protein family as the LSK109 sequencing chemistry. This adapter has a significant disadvantage for short fragment libraries because it incurs active consumption of on-chip “fuel” of idle sequencing molecules, leading to rapid flow cell exhaustion. To address this issue, we modified the library preparation process to incorporate the updated “fuel-fix” adapter (LSK110 kit, Oxford Nanopore Technologies), which at the time when these experiments were conducted, did not have multiplexing capabilities. We developed a protocol to enable multiplexing with this adapter. We performed a second end-repair and A-tailing reaction using the Kapa HyperPrep library preparation kit. This step removed the sticky end from the barcode multiplexing adapter and produced a compatible A-tail for sequencing adapter ligation. We used an increased amount (10 μl) of the AMX-F sequencing adapter (LSK110, Oxford Nanopore Technologies) for the ligation step to maximize the yield of sequencing adapters to barcoded fragments. This second ligation reaction occurred for 1.5 h. Subsequently, we mixed in 88 μl of Mag-Bind Total NGS beads and incubated for 5 min. As in the standard protocol, we washed the beads with 200 μl SFB buffer (Oxford Nanopore Technologies) with gentle tube flicking to resuspend the beads during the wash steps. The beads were resuspended in EB buffer (Oxford Nanopore Technologies). We used 1 μl for quantification with Qubit (Oxford Nanopore Technologies) and 1 μl for determining the DNA size with an E-gel EX cartridge (Thermo Fisher Scientific).

We generated sequencing libraries for tumor tissue and PBMCs with 1–2 μg of extracted genomic DNA. For some tissue and buffy coat samples with low extraction yields (less than 1 μg), we used the entire amount of extracted DNA for library preparation. We followed the standard Kapa HyperPrep library preparation kit protocol using 5 μl of AMX-F adapter (LSK110) without barcoding. Each sample was loaded into its own PromethION flow cell for sequencing. For comparison with the standard library preparation protocol, we followed the standard protocol for Native Barcoding (EXP-NBD196) coupled with the SQK-LSK109 library preparation kit using the AMII adapter. The standard protocol is available on the Oxford Nanopore Technologies website.

### Nanopore sequencing and data processing

We performed sequencing on the Oxford Nanopore Technologies’ PromethION 24 instrument using R9.4.1 PromethION flow cells. After quantification and sizing of the final pooled library, we calculate its molarity. We split the library among multiple flow cells depending on its final concentration. Approximately 150fmol of the library was loaded for each flow cell, loading up to four flow cells in a single batch. The remainder of the library was stored at − 80 °C. For tissue samples, we used one entire flow cell per sample. Sequencing runs had a duration of 72 h. Barcode demultiplexing was performed on the sequencer using onboard basecalling in MinKNOW with the “high accuracy” model and then transferred to a separate storage device. Raw demultiplexed fast5 sequencing data were processed using Megalodon v2.4.0 (Oxford Nanopore Technologies, https://github.com/nanoporetech/megalodon) and Guppy v5.0.16 (Oxford Nanopore Technologies, available closed source at https://nanoporetech.com/) with the “dna_r9.4.1_450bps_modbases_5mc_hac_prom.cfg” model for each demultiplexed barcode folder with standard settings. The quality score cutoff was 7. The GRCh38 reference was used for alignment. The output consists of a file in BedMethyl format for each sample. The files included modified base calls, a sequencing alignment bam file with modified base calls for each read, and a per-read text file containing modified base call probabilities. Before further processing, the BedMethyl and sequence alignment bam files were sorted and indexed with samtools [13]. For larger sequencing runs involving multiple samples (e.g., from multiple flow cells and many barcodes), data was transferred to the Sherlock High-Performance Computing cluster at Stanford University for multi-node GPU-based data processing.

The overall methylation status of sequenced cfDNA was determined by taking the average of all methylation values across all sequenced sites that had at least one read (coverage > 0). To determine nucleosome enrichment, we subsampled each library’s sequence-aligned bam file to 50,000 reads, tabulated the estimated fragment size as inferred by the alignment length, and set a cutoff of 250.5 base pairs separating mono- and di-nucleosome states, with a maximum length filter of 600 bp. This data was then compiled for all reads and all samples sequenced.

We determined gene-level methylation for all sequenced cfDNA samples by calculating the average per-site methylation for each CpG site with non-zero coverage, and then searched for statistically significant differences in gene-level methylation. This procedure is similar to that of another study [7], with the main difference being that our data enables site-level detection of methylation percentage. We utilized “gene”-level annotations in GENCODE v38 [14], which includes all coding exons and introns. First, we filtered on genes which were covered by at least one sample and where the standard deviation of average gene methylation is greater than zero. Then, grouping by gene-level annotations, we calculated the average methylation. Based on genomic coordinates, we then excluded annotations that were pseudogenes, unprocessed, “to be experimentally confirmed genes,” lncRNAs, and miRNAs. Finally, we used a *t*-test to compare methylation between the healthy donor-derived cfDNA and cancer patient-derived cfDNA. An FDR-based multiple testing correction was applied to determine statistically significant differences in gene-level methylation. We used a cutoff of *q* < 0.01. For promoter region analysis, we selected a window beginning 2 kb upstream and 500 bp downstream of a gene annotation while maintaining strand specificity.

### In silico admixture analysis

To simulate circulating tumor DNA (ctDNA) data of varying fractions in cfDNA, we generated in silico admixtures of sequence data from the GP2D cancer cell line-derived and PBMC-derived nucleosomes. Using a Python script, we mixed two sequence-aligned bam files using a known random seed to ensure reproducibility. We also controlled for the number of reads to simulate different read depths. Methylation profiles were compiled from the Mm and Ml tags using the modbampy library as part of the modbam2bed package (https://github.com/epi2me-labs/modbam2bed). We used only reads that mapped to the reference and used the subsequent bam file for downstream analysis. The remainders of the reads were not used, including unmapped reads and those with secondary or supplementary alignments. As another output, we included the metadata about the sample origins, namely whether it originated from PBMC-derived nucleosomes or a cancer cell line.

### Reference methylation profile processing of tumors and immune cells

For a subset of the patient samples, we had matched tumor tissue and PBMCs (three patients: P6199, P4822, and P6527). These matched samples underwent nanopore sequencing to generate reference methylomes. Methylation calls were also performed with megalodon. The reference methylomes consist of megalodon’s output BedMethyl files, which contain genomic positions of CpG sites, coverage (> 0), and the associated percent methylation for that position.

To process these reference profiles for read-level classification, we used an R script to read both the tumor and PBMC methylation profiles. We intersected these profiles on genomic coordinate positions, with a coverage filter of greater than four in both samples [15]. We considered a site to be methylated if the percentage methylation per a given genomic segment was greater than zero. The resultant intersected table was used for read-level classification.

To determine gene-level methylation for primary tumor and blood samples, average methylation profiles were determined for each “gene”-level annotation in GENCODE v38 [14]. These were then filtered to only include genes with the annotation “protein_coding.” We calculated the difference in methylation between the primary tumor and immune cells, and selected the top 25 genes for each methylation state (e.g., top 25 differentially hypermethylated genes, and top 25 differentially hypomethylated genes). We also ensured that the primary tumor and blood sample have opposing methylation stages (e.g., hypermethylated vs hypomethylated) by requiring that for any annotation the tumor sample must have over 50% methylation and the blood sample must have less than 50% methylation, or vice versa. Using this gene list, we extracted the methylation values from the patient cohort that underwent longitudinal sampling.

### Single-molecule read classification to reference profiles

We built a computational workflow to classify whether an individual read is associated with an associated reference methylation profile. It consists of two steps:

- 1. Read-level methylation processing: This process utilizes sequence-aligned bam files containing read modifications (from megalodon). We used a python script to emit a table with columns consisting of the read name, genomic coordinate, and called methylation status. The output is a flat data table whereby genomic coordinates and their methylation states can be grouped by individual reads.
- 2a. Scoring against reference profiles: We classified each read alongside a reference methylome containing informative methylation sites. Reference methylomes consist of matched tumor and immune cell methylation profiles that were nanopore sequenced and processed as above. Informative sites are CpG sites where the methylation values differed between sample types. This process generated a value ${f}_{i}^{\mathrm{tissue}}$ where *i* is the read number from 1 to the total number of aligned reads.
- Specifically, $${f}_{i}^{\mathrm{tissue}}={\sum }_{j}^{{N}_{\mathrm{sites}}}prob\left({m}_{j}={m}_{j}^{^{\prime}}\right)/{N}_{\mathrm{sites}}=\left\{\begin{array}{c}{m}_{j}^{^{\prime}}\ \ if \ \ {m}_{j}=1\\ 100-{m}_{j}^{^{\prime}}\ \ if \ \ {m}_{j}=0\end{array}\right.$$ where *i* is the read number, *tissue* is the candidate reference profile to match against, *m*_*j*_ is the methylation of read *i* at site* j* (either 0 or 1),* m*_*j*_*’* is the methylation of *tissue* at site *j* (ranging from 0 to 100), and *N*_sites_ is the number of methylated sites to consider for read *i*.
- In other words, ${f}_{i}^{\mathrm{tissue}}$ is the mean probability that read *i* matches to a specific *tissue* methylation reference profile. To implement this scheme, we obtained the methylation status (*m*_*i*_* … m*_*n*_) of each CpG site for each read from a given sample and its reference coordinate. Then we intersected these coordinates of the CpG sites to the corresponding locations of a candidate tissue reference methylation profile (e.g., from PBMCs or matched primary tumor, with methylation profile *m*_*i*_*’ … m*_*n*_*’*). Subsequently, we calculated a matching score, where each site is scored *m*_*i*_*’* if the *m*_*i*_ is methylated; otherwise, it is scored 100 − *m*_*i*_*’*. In other words, the score is the probability that the methylation site and value *m*_*i*_ is the same as the reference profile site *m*_*i*_*’*, which is equivalent to the reference profile’s methylation level at that site. It is then divided by the total number of candidate CpG sites on the read to derive ${f}_{i}^{\mathrm{tissue}}$. Reads with no candidate CpG sites or matching locations in a reference methylome were not considered.
- 2b. Score normalization and thresholding: A normalized per-read tumor score was then assigned by the ratio of scores ${p}_{i}={f}_{i}^{\mathrm{tumor}}/({f}_{i}^{\mathrm{tumor}}+{f}_{i}^{\mathrm{immune}})$, with scores close to zero indicating likely matches to PBMCs, and scores close to one indicating likely matches to tumor tissue. A final classification is determined by setting thresholds for matching to PBMC and cancer methylation profiles. By using a dual threshold system, a subset of reads in between the thresholds do not have a confident/stringent classification and were not called to be either type (and thus can be neglected from the final analysis). We varied the two thresholds to determine ROC curves and AUC performance metrics.
- 3. Processing all reads. We process and aggregate classification calls from all reads in order to calculate the fraction of reads with methylation changes that were classified as specific to the tumor.

## Results

### Enabling order-of-magnitude improvements in nanopore sequencing yield

Nanopore sequencing typically requires hundreds of nanograms of DNA for library preparation. However, extracted cfDNA yields range from single nanograms or less per ml of plasma. PCR amplification erases DNA methylation and cannot be used for nanopore-based methylation analysis. To enable PCR-free library preparation from nanogram DNA amounts of cfDNA, we identified a series of steps to efficiently incorporate sample barcodes and nanopore sequencing adapters to cfDNA (Additional file 1: Supplementary Fig. 1). We systematically optimized reaction conditions to maximize cfDNA library yield. The method also incorporated a second end-repair and a-tailing step to add newer sequencing adapters for multiplexing cfDNA samples (Methods).

To validate our approach, we developed a model DNA analyte that replicates some of the fragmentation patterns of cfDNA. We performed DNase digestion on nuclei from isolated peripheral blood mononuclear cells (PBMCs), where digestion of open chromatin yields DNA fragmentation patterns with a mononucleosome peak as seen in cfDNA (Additional file 1: Supplementary Fig. 2). With this DNA, we used our library preparation method to generate libraries that were sequenced on the Oxford Nanopore PromethION system. We used the “megalodon” software package, previously benchmarked to provide high-quality methylation calls from nanopore sequence data concordant with traditional bisulfite sequencing [10, 11], for sequence alignment and methylation calling (Additional file 1: Supplementary Fig. 3). Specifically, nanopore sequencing was previously benchmarked to have a Pearson correlation of over 0.9 with whole-genome bisulfite sequencing data [10, 11]. We sequenced libraries made from different amounts of PBMC nucleosomal DNA. At 5 ng of input DNA, the yield was approximately 6 million aligned reads inclusive of quality score filtering. With 100 pg of input DNA, the yield was approximately 140,000 aligned reads (Additional file 1: Supplementary Table 1). We also prepared sequencing libraries of the same DNA with a standard protocol from Oxford Nanopore Technologies. Our method improved the aligned read yield by approximately an order of magnitude, even with input amounts as low as 100 pg (Fig. 1B).

### Cell-free DNA methylation patterns in a colorectal cancer cohort

Next, we sequenced cfDNA from 20 patients with colorectal cancer (Additional file 1: Supplementary Table 1, 2). Sequence yields ranged from one to 180 million reads per sample. We used a fluorometric assay to quantify the cfDNA of each sample (Fig. 1C); the measurements were highly correlated with the total sequencing yield (Spearman’s rho = 0.86, *p* < 2.2e − 16). As a control, we sequenced cfDNA from healthy individuals. There were several significant differences when comparing healthy and cancer patient cfDNA. First, the overall variance in genome-wide methylation in cancer patient cfDNA samples was higher at 7% compared to less than 2% in healthy cfDNA (Fig. 1D). The variation may be indicative of aggregate methylation shifts due to an increase in tumor-specific cfDNA in plasma, although larger numbers of healthy controls and cancer patients are needed to make a statistically powered conclusion.

Next, we sought to determine whether there are any statistically significant changes in cfDNA fragment size distributions between cancer patients and healthy controls. We distinguished mono- and di-nucleosome fragments using a size cutoff of 250 bp (Fig. 1E, F, Additional file 1: Supplementary Fig. 4). We observed that cancer patient cfDNA was enriched in mononucleosomes by approximately a factor of two (*p* = 8.814e − 05) compared to healthy controls. A similar result was also reported in another study [16].

We next determined the extent of gene-level methylation in cfDNA (Methods). To do so, we calculated the average per-site methylation for each CpG site with non-zero coverage, and then filtered for statistically significant differences in gene-level methylation between cancer patients and healthy controls. We observed many significant differences in gene-level methylation (*q* < 0.01) when comparing healthy versus patient cfDNA (Fig. 1G, H, Additional file 1: Supplementary Table 3). For example, there was an increase in the methylation of an immunologic marker gene *CD79A* [17], a decrease in methylation of a tumorigenic modulation gene *DICER1* [18], and a decrease in methylation of *SLC25A1*, a critical gene in mitochondrial homeostasis that is highly upregulated in cancer [19, 20]. Due to differences in biological relevance between promoter-level and gene-level methylation [21–24], we also performed an additional analysis to discover statistically significant differences in promoter-level methylation (Additional file 1: Supplementary Table 4). Top statistically significant hits included *ADAMTS5*,* ATP6V1C2*, and* S100A6*—genes that have been previously identified as biomarkers for colorectal cancer [25–28].

We further examined the genes with statistically significant methylation differences between healthy controls and patient cfDNA. We calculated that such differences were not strongly correlated (Pearson *r* =  − 0.166, *p* = 3e − 13) with the sequencing read fold-coverage for those genes (Additional file 1: Supplementary Fig. 5A,B). This result indicated that read yields were not a major confounding factor in our analysis and that deep sequencing is not necessarily to distinguish cfDNA methylation between cancer patients and healthy controls. We also observed that the overall variation in gene-level methylation for these statistically significant genes was comparatively smaller in healthy cfDNA samples, indicating a relatively uniform methylome for this sample type (Additional file 1: Supplementary Fig. 6A). We also observed a relatively uniform cfDNA methylome across healthy cfDNA samples compared to cancer patients’ cfDNA (*p* = 4.57e–4), further indicating that statistically significant methylation changes point to a biological difference rather than sample-to-sample variability. To further validate that our results pointed to biological variation rather than as a result of sample-to-sample variability, we repeated our statistical analysis for finding differentially methylated genes but instead using random grouping of samples. We randomly assigned the combined cancer patient and healthy individual cfDNA cohort into two groups and performed the same differentially methylated gene analysis as before. After testing for statistically significant differences and FDR-based multiple testing correction, we found that zero genes passed the *q* < 0.01 filter (Additional file 1: Supplementary Fig. 6B). This procedure was repeated 20 times. This suggests that differences that came from our analysis were due to biological variation rather than by chance.

Finally, enrichment analysis of genes with statistically significant methylation differences using EnrichR [29, 30] yielded hits in the Myc pathway (Additional file 1: Supplementary Fig. 7A), strongly indicating that the changes in gene-level methylation point to a cancer-specific biological mechanism. Other potential hits, which were not statistically significant, include E-cadherin pathways that are commonly implicated in epithelial cancers and not healthy immune cells (Additional file 1: Supplementary Fig. 7B). Promoter-level differential methylation analysis also yielded enrichment in the tumorigenic Ras pathway but also did not reach statistical significance (Additional file 1: Supplementary Fig. 7C,D). We also observed differences in the overall methylome profiles of individual cancer patients; however, disentangling these intra-cohort differences requires significant knowledge of other factors such as treatment status and molecular subtype which are beyond the scope of this study.

### Single-molecule classification of cell-free DNA sequence reads

**Fig. 2:**
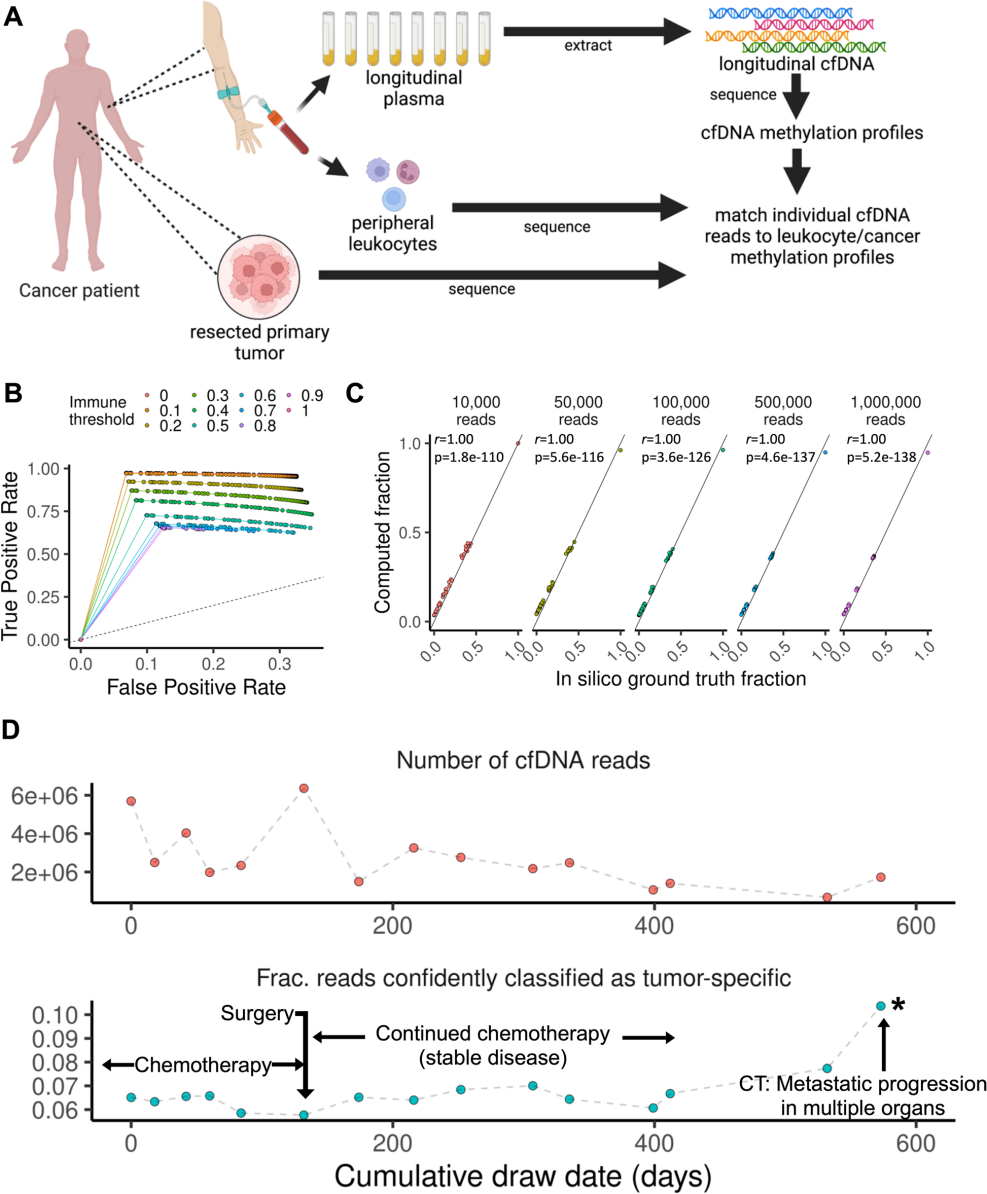
Single-molecule methylated sequence classification. **A** Overview of method. For a given patient, matched primary tumor tissue and peripheral leukocytes were obtained as reference samples alongside longitudinal plasma samples. Methylation data from the cfDNA is then classified leveraging the methylation profile of the reference samples. **B** Classification accuracy. We used GP2D and healthy donor-derived nucleosome mixtures to validate the classification procedure. ROC curves are plotted, where each curve represents a distinct immune threshold score. The curve is plotted by varying the cancer threshold score. **C** Admixture validation. The proportion of reads classified as belonging to cell line reference is plotted as a function of the actual admixture ratio and sequencing depth. **D** Longitudinal methylation profiles of patient-derived cfDNA in colorectal cancer. The overall cfDNA sequencing yield (upper panel) is plotted against the number of reads with methylation profiles matching that of the matched tumor with a calculated score of > 0.9 (lower panel). Clinically relevant events are annotated; significant corresponding changes in tumor-specific cfDNA are marked with an asterisk. CT refers to computed tomography imaging

We determined whether individual reads from cfDNA sequence data can be classified as originating from tumor or immune cells. We leveraged patient-matched samples, which included resected tumors, peripheral leukocytes, and blood samples taken during treatment (Fig. 2A). DNA was extracted from these matched samples and underwent nanopore sequencing with methylation profiling. To classify each read, we calculated the proportion of matching methylation sites based on genomic coordinate and methylation states when compared to the matched tumor or immune cell methylation profile. In other words, we measure for each individual read (e.g., from cfDNA) how similar its methylation profile is to another reference sample’s methylation profile (e.g., a primary tumor or immune cells) at the same alignment location. For each read, we intersected their aligned CpG coordinates alongside their methylation status with those of each reference sample. The proportion of matching methylation sites is then calculated for each read. The result is a classification score for each read which can then be aggregated into an overall proportion for all reads in a given cfDNA sample (Additional file 1: Supplementary Fig. 8). After normalization, thresholds were used to classify individual reads as immune cell- or tumor-derived (Methods, Additional file 1: Supplementary Fig. 9). Example read pileups of this process as well as example classifications are shown in Additional file 1: Supplementary Fig. 10.

We validated this approach using in silico admixtures between digested PBMC nucleosomes from a healthy donor and the GP2D cancer cell line. Nucleosomal DNA was subject to high-depth nanopore sequencing and methylation calling. Admixtures were computationally generated, and their proportions were estimated using our classification approach (Methods). We measured the classification accuracy against the ground truth based on the source of any given read (Fig. 2B). Performance was up to 97% sensitivity and specificity of 93% with an AUC of 0.951 when using stringent threshold cutoffs (immune threshold: < 0.1, cancer threshold: > 0.9, Additional file 1: Supplementary Fig. 11). There was a corresponding trade-off with a declining proportion of classified reads (Additional file 1: Supplementary Fig. 12); reads that could not be confidently classified as either type would be excluded from further analysis. Gene set enrichment of regions that were substantially different and were most predictive of sample type were highly enriched in epithelial cytoskeletal remodeling and proliferation pathways (Additional file 1: Supplementary Fig. 13A,B). We also simulated varying number of reads and did not observe declines in quantification performance when using stringent cutoffs (Fig. 2C).

We also generated experimental admixtures where GP2D nucleosome DNA was added to donor nucleosome DNA while also varying the total quantity of DNA in the reaction. We observed a corresponding increase in cancer-derived reads at higher GP2D admixture fractions with Pearson correlation coefficients, ranging from 0.85 to 0.96 depending on the input amount (Additional file 1: Supplementary Fig. 14).

### Longitudinal assessment of tumor burden with nanopore sequencing

For three patients (P4822, P6199, P6527) with different gastrointestinal cancers, we analyzed cfDNA from a longitudinal series of blood samples. The methylation profiles revealed responses to specific treatments and the emergence of treatment-resistant metastatic cancer. We performed nanopore sequencing on the set of patient-matched tumor, peripheral blood, and longitudinal plasma samples and determined their methylation profiles (Fig. 2A, Additional file 1: Supplementary Table 1,2). Matched tumor and immune cells were sequenced up to 28 × coverage. Their methylomes, intersected by genomic position and filtered by coverage, yielded tens of millions of CpG sites per patient.

We observed longitudinal trends that correlated with specific clinical events based on our analysis. For one patient (P6199) receiving treatment for metastatic colorectal cancer, we sampled blood over approximately 600 days (Fig. 2D). After having undergone chemotherapy and surgery, the patient had a period of stable disease. However, starting after day 400, the fraction of reads with tumor-specific methylation changes dramatically increased. This change correlated with CT imaging which showed substantial metastatic progression in multiple organs. We also sought to determine overall gene-level methylation changes. As a baseline for comparison, we considered informative genes with differential methylation between tumor and immune cells as having the largest differences in overall methylation levels. Gene-level methylation analysis of this patient’s longitudinal cfDNA showed dynamic gene-level methylation changes also found in the matched tumor, such as the Wnt/β-catenin regulator *TCIM* [31] (Additional file 1: Supplementary Fig. 15A,B).

**Fig. 3:**
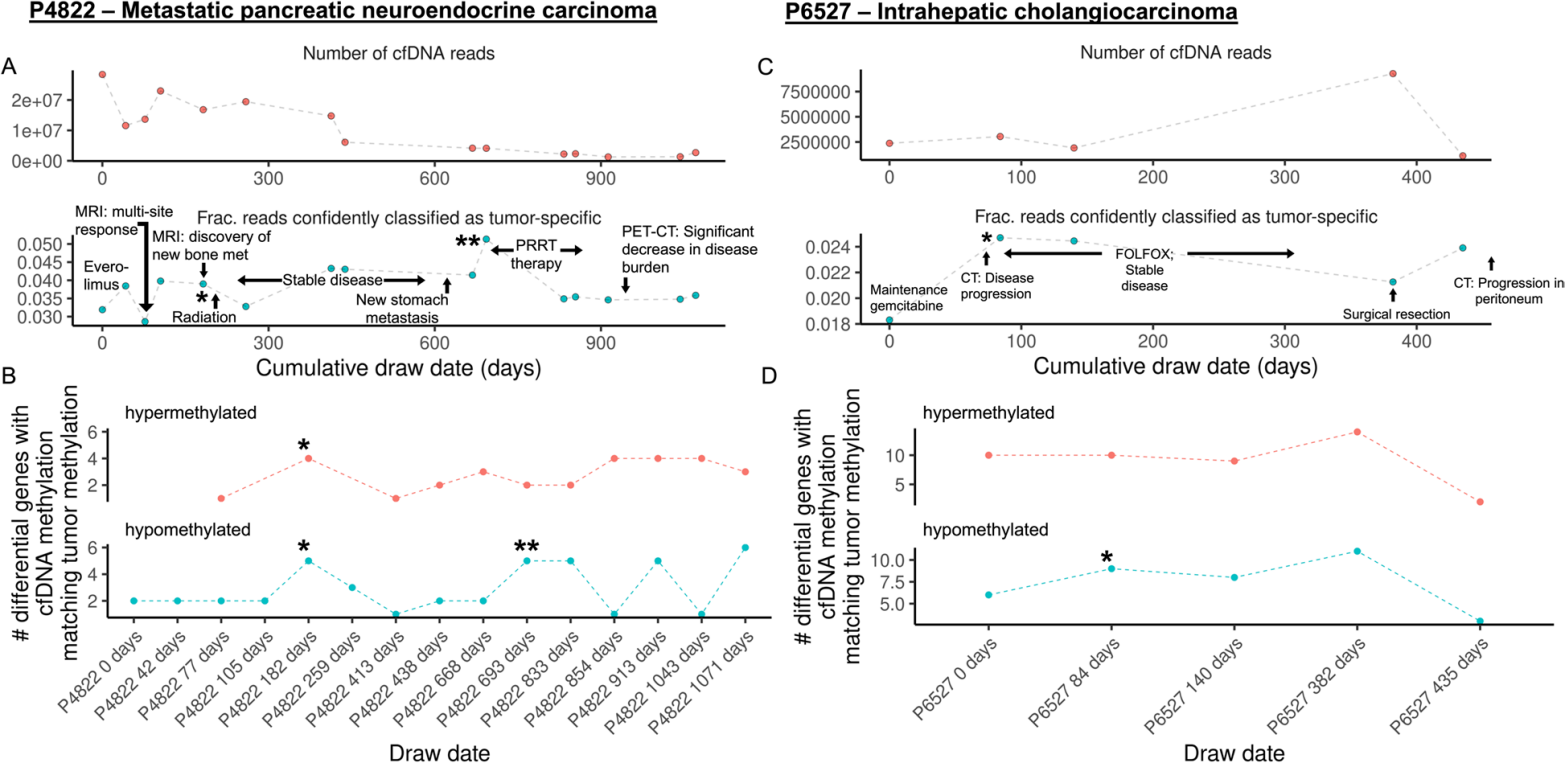
Longitudinal methylation profiles of patient-derived cfDNA in other malignancies. **A** Assessing tumor burden in patient P4822 with metastatic pancreatic neuroendocrine carcinoma. The overall cfDNA sequencing yield (top) is plotted against the number of reads with methylation profiles matching the primary tumor with a tumor score of > 0.9 (bottom). Clinically relevant events are annotated. Clinically relevant events are annotated. Everolimus and peptide receptor radionuclide therapy (PRRT) was used for treatment of the metastatic neuroendocrine cancer. Positron-emission tomography (PET) was combined with CT imaging. **B** Longitudinal gene-level analysis of cfDNA changes in P4822. The number of tumor-specific differentially methylated genes found to be matching in cfDNA is shown for each time point. Differentially methylated genes were identified as those with the largest difference in methylation between the primary tumor and immune cells. Such methylated genes observed in cfDNA are defined as matching the primary tumor when its methylation state (e.g., hypermethylation or hypomethylation) is concordant. Specific time points are annotated with asterisks to denote clinical events with significant changes in methylation. **C** Assessing tumor burden in patient P6527 with metastatic cholangiocarcinoma. The overall cfDNA sequencing yield (top) is plotted against the number of reads with methylation profiles matching the primary tumor with a tumor score of > 0.9 (bottom). Clinically relevant events are annotated. Treatment included gemcitabine and a chemotherapy combination of 5-fluouracil and oxaliplatin (FOLFOX) was used for treatment of the cholangiocarcinoma. **D** Longitudinal gene-level analysis of cfDNA changes in P6527. The number of tumor-specific differentially methylated genes found to be matching in cfDNA is shown for each time point. Differentially methylated genes were identified as those with the largest difference in methylation between the primary tumor and immune cells. Such methylated genes observed in cfDNA are defined as matching the primary tumor when its methylation state (e.g., hypermethylation or hypomethylation) is concordant. Specific time points are annotated with asterisks to denote clinical events with significant changes in methylation

We performed similar analyses on two other patients with metastatic cancer. One patient (P4822) had metastatic pancreatic neuroendocrine carcinoma and received multiple treatments, including targeted therapy, radiation, and peptide receptor radionuclide therapy (PRRT) spanning over 1000 days (Fig. 3A). After each treatment, there was a correlation between treatment effect and a drop in tumor-specific reads. The emergence of new metastases was reflected in a rise in reads with tumor-specific methylation changes. Similar to patient P6199, increases in tumor-specific reads for various clinical events were correlated with some gene-level changes in methylation. Specifically, we observed cfDNA methylation changes in tumor-specific differentially methylated genes (Fig. 3B, Additional file 1: Supplementary Fig. 16). For another patient (P6527) with metastatic cholangiocarcinoma, resistance to the initial chemotherapy with gemcitabine was evident, but disease was reduced under a dual chemotherapy treatment (Fig. 3C). Disease progression was noted up to 100 days into the study and was confirmed by both tumor-specific read counts and changes in tumor-specific gene-level methylation (Fig. 3D, Additional file 1: Supplementary Fig. 17). The patient underwent extensive surgical resection of the primary tumor and liver metastases, as reflected in the immediate drop in tumor-specific reads. However, a subsequent rise in tumor-specific reads coincided with metastatic cancer recurrence.

## Discussion

We demonstrated a single-molecule approach for efficiently analyzing methylomes from cfDNA at scale. Improving on existing approaches by approximately an order of magnitude, our method yielded over 1 billion total aligned reads across our cancer cohort when using small amounts of cfDNA. We detected cancer-associated methylation profiles with distinguishing epigenetic characteristics and provided a measurement of tumor burden that corresponded to clinical events.

Our work demonstrated streamlined methylation analysis of cfDNA with significantly fewer experimental procedures and bottlenecks than short-read sequencing (Additional file 1: Supplementary Table 5). Specifically, other cfDNA sequencing workflows may involve treatment of canonical cytosine conversion reagents such as bisulfite and require target enrichment and PCR steps, and extensive assay cleanups that can reduce yield. Several studies investigating whole-genome bisulfite sequencing have identified significant issues that may impact data quality [9, 32], including DNA degradation after cytosine conversion, substantial amplification bias during PCR, and incomplete conversion of canonical cytosine bases. Our workflow is a ligation-based protocol that can be completed with less than 8 h of total hands-on time with a single overnight incubation step, which effectively reduces the potential for sample loss. Despite the lack of PCR amplification, our method robustly captures methylation profiles of cfDNA; improvements in nanopore sequencing chemistry as well as utilization of larger amounts of plasma are anticipated to substantially increase data yields. Although originally conceived as a long-read sequencing platform, we demonstrated that nanopore sequencing could be robustly used for small cfDNA fragments with significant potential for clinical utility. In particular, our study shows clear potential for large cfDNA cohorts to be nanopore sequenced, thus potentially enabling new applications in the area of cancer screening.

Methylation detection is performed through the use of machine learning models on raw electrical signals from nanopore sequencing data. By archiving this data, future improvements in basecalling and machine learning models can be leveraged to detect other types of epigenetic biomarkers. Newer machine learning models that incorporate the detection of other modified bases such as 5-hydroxymethylcytosine (5hmC) methylation can be applied in future studies to further explore cfDNA methylation profiles in cancer without performing more experiments on precious material.

We also demonstrated a proof-of-concept implementation of a single-molecule methylation-based classifier using matched tumors and blood as reference profiles. It relies on detecting statistically significant differences in methylation between samples. To validate this approach, we used admixtures of nucleosomes from healthy immune cells and cancer cell lines. While this system provides a clean model for ascertainment of cancer quantification, methylation profiles in primary tumors are confounded by intratumoral heterogeneity and mixtures with other cell types. This can impact the extent to which individual molecules can be successfully classified. Nonetheless, as a proof-of-concept application to demonstrate possible clinical utility, we monitored cfDNA methylation dynamics over the course of treatment in a number of patients, with cancer-specific cfDNA molecules correlating with clinical events such as tumor response and recurrence. As a further area of study, more time points and more plasma will need to be sequenced to further validate the clinical utility of the longitudinal approach. We also envision that this approach can also be applied for patients receiving treatment without a matched tumor, provided a reference methylome database of immune cells, normal tissues, and primary tumors can be used to match individual cfDNA reads. Specifically, genomic DNA from healthy blood and primary tumors of specific cancer types could be sequenced. After determining statistically significant differences, nanopore sequencing could be performed on cancer patient cfDNA and then quantified for the extent of tumor burden. We also envision that reference methylome datasets would also be immensely useful for disentangling cell type-specific methylation versus tumor-specific methylation in liquid biopsy applications.

Recently, another publication demonstrated the use of nanopore sequencing for analyzing cfDNA methylomes in cancer patients [33]. It demonstrated significant capabilities in nucleosome footprinting, cell-of-origin determination, and integration with copy number profiling using nanopore sequencing. Our work is distinct in several ways. First, we developed significant advances in sequencing capacity by improving library yields by up to an order of magnitude—this enables robust sequencing yields from less than a milliliter of plasma. Current methods typically use at least 4–5 ml of plasma (an entire blood collection tube) for extraction of cfDNA material. Second, we leveraged high-throughput sequencing on the PromethION instrument to enable cohort-level sequencing of cancer patient cfDNA alongside healthy controls. Third, we built a single-molecule classifier for tumor burden quantification, versus bulk-level deconvolution. Lastly, we leveraged all of these innovations for comprehensive longitudinal monitoring in cancer patients for up to 1000 days. Nonetheless, that particular work and our work are complementary and demonstrate the potential for nanopore-based methylome characterization to advance the liquid biopsy field.

## Conclusions

In summary, we describe a single-molecule sequencing method that enables the analysis of cfDNA methylation. We improved sequencing yields by an order of magnitude, applied it to cohort-level analyses, and developed a single-molecule classifier. We also applied these methods to longitudinally monitor cancer treatment. This approach has the potential to impact liquid biopsy diagnostics for cancer detection and characterization.

## Supplementary Information

**Additional file 1:** **Supplementary Figure 1.** Sequencing library preparation workflow. **Supplementary Figure 2.** Digested nucleosome size comparison with cfDNA. **Supplementary Figure 3.** Computational workflow. **Supplementary Figure 4.** Fragment size distribution of healthy donor and cancer patient cfDNA. **Supplementary Figure 5.** Correlation between gene-level fold coverage and gene-level methylation. **Supplementary Figure 6.** Analysis of variability in gene-level methylation. **Supplementary Figure 7.** Enrichment analysis for cancer patient cohort. **Supplementary Figure 8.** Framework for read classification. **Supplementary Figure 9.** Distribution of tumor classification scores for single reads. **Supplementary Figure 10.** Examples of read classification. **Supplementary Figure 11.** Classification AUC for various thresholds. **Supplementary Figure 12.** Fraction of reads classified. **Supplementary Figure 13.** Gene enrichment analysis for single molecular classifier using a GP2D cancer cell line and immune cell model. **Supplementary Figure 14.** Experimental admixtures. **Supplementary Figure 15.** Gene-level visualization for longitudinally collected plasma samples for patient P6199. **Supplementary Figure 16.** Gene-level visualization for patient P4822 with metastatic pancreatic neuroendocrine carcinoma. **Supplementary Figure 17.** Gene-level visualization for patient P6527 with metastatic cholangiocarcinoma. **Supplementary Table 1.** Sequencing Metrics. **Supplementary Table 2.** Patient Information. **Supplementary Table 3.** Genes with significant methylation differences between healthy and patient-derived cfDNA in 20 patient cohort. **Supplementary Table 4.** Promoter regions with significant methylation differences between healthy and patient-derived cfDNA in 20 patient cohort. **Supplementary Table 5. **Methods Comparison.

## Data Availability

Sequence-aligned BAM files and associated methylation calls are deposited in NCBI’s dbGaP under accession phs002950 at the URL https://www.ncbi.nlm.nih.gov/projects/gap/cgi-bin/study.cgi?study_id=phs002950 [34]. Code is available at https://github.com/billytcl/nanopore_cfDNA [35].

## Abbreviations

5mC: 5-Methylcytosine
5hmC: 5-Hydroxymethylcytosine
cfDNA: Cell-free DNA
ctDNA: Circulating tumor DNA
PBMC: Peripheral blood mononuclear cell
PRRT: Peptide receptor radionuclide therapy
