# Supplementary material for: Single-molecule methylation profiles of cell-free DNA in cancer with nanopore sequencing
Source: Genome Med. 2023 May 3;15:33. doi: 10.1186/s13073-023-01178-3 (PMC10155347; doi:10.1186/s13073-023-01178-3)
Supplement: Supplementary file 1 — Additional file 1: Supplementary Figure 1. Sequencing library preparation workflow. Supplementary Figure 2. Digested nucleosome size comparison with cfDNA. Supplementary Figure 3. Computational workflow. Supplementary Figure 4. Fragment size distribution of healthy donor and cancer patient cfDNA. Supplementary Figure 5. Correlation between gene-level fold coverage and gene-level methylation. Supplementary Figure 6. Analysis of variability in gene-level methylation. Supplementary Figure 7. Enrichment analysis for cancer patient cohort. Supplementary Figure 8. Framework for read classification. Supplementary Figure 9. Distribution of tumor classification scores for single reads. Supplementary Figure 10. Examples of read classification. Supplementary Figure 11. Classification AUC for various thresholds. Supplementary Figure 12. Fraction of reads classified. Supplementary Figure 13. Gene enrichment analysis for single molecular classifier using a GP2D cancer cell line and immune cell model. Supplementary Figure 14. Experimental admixtures. Supplementary Figure 15. Gene-level visualization for longitudinally collected plasma samples for patient P6199. Supplementary Figure 16. Gene-level visualization for patient P4822 with metastatic pancreatic neuroendocrine carcinoma. Supplementary Figure 17. Gene-level visualization for patient P6527 with metastatic cholangiocarcinoma. Supplementary Table 1. Sequencing Metrics. Supplementary Table 2. Patient Information. Supplementary Table 3. Genes with significant methylation differences between healthy and patient-derived cfDNA in 20 patient cohort. Supplementary Table 4. Promoter regions with significant methylation differences between healthy and patient-derived cfDNA in 20 patient cohort. Supplementary Table 5. Methods Comparison. [file 13073_2023_1178_MOESM1_ESM.pdf]

SUPPLEMENTARY INFORMATION

TITLE

Single molecule methylation profiles of cell-free DNA in cancer with nanopore sequencing

AUTHORS

Billy T. Lau[1], Alison Almeda, Marie Schauer[1], Maddy McNamara[1], Xiangqi Bai[1], Qingxi Meng[2], Mira Partha[2], Susan M. Grimes[1], HoJoon Lee[1], Gregory M. Heestand[1], Hanlee P. Ji[1,2]

[1]Division of Oncology, Department of Medicine, Stanford School of Medicine, Stanford CA

[2]Department of Electrical Engineering, Stanford University, Stanford CA

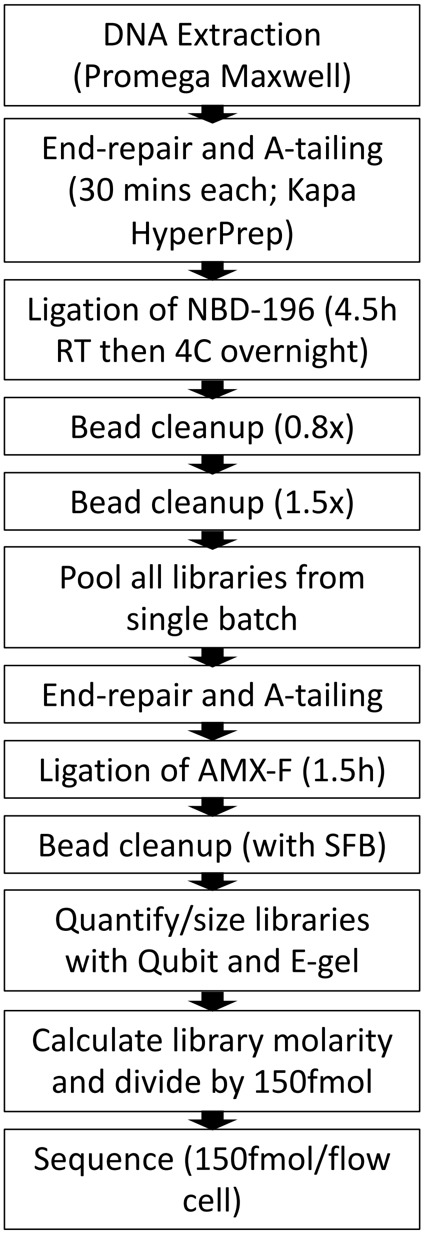

Supplementary Figure 1. Sequencing library preparation workflow. The library preparation workflow used in this study for cfDNA samples. Sequencing was conducted on the Oxford Nanopore platform. These steps maximized ligation yields versus standard protocols.

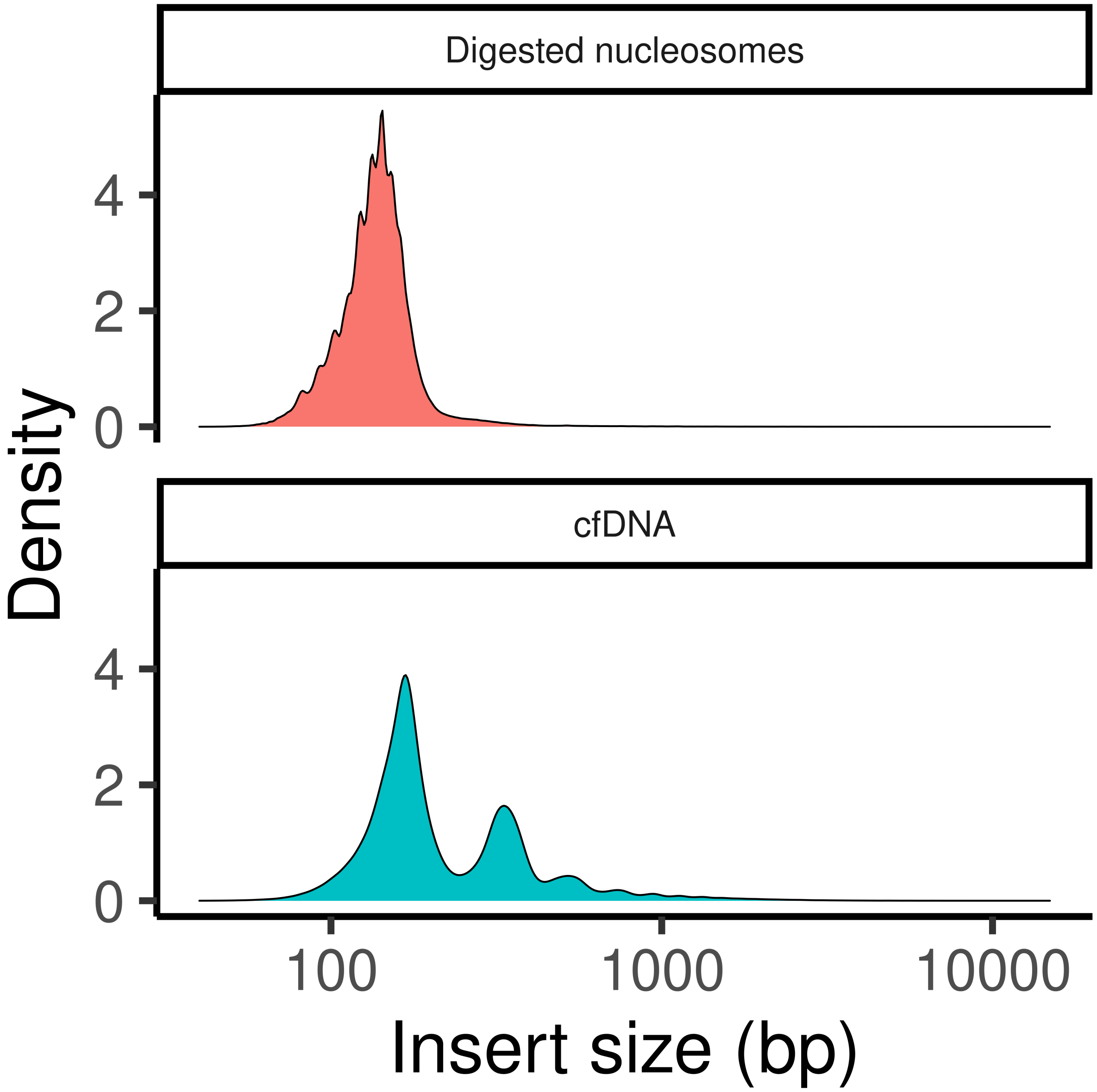

Supplementary Figure 2. Digested nucleosome size comparison with cfDNA. The insert size distribution of digested PBMC nucleosomes (top), which was used as a model analyte. This size distribution was compared to the size distribution of cfDNA. Secondary peaks in the cfDNA distribution correspond to dinucleosomes and higher sizes. The PBMC nucleosomes consisted only of mononucleosomes due to complete digestion of the open chromatin.

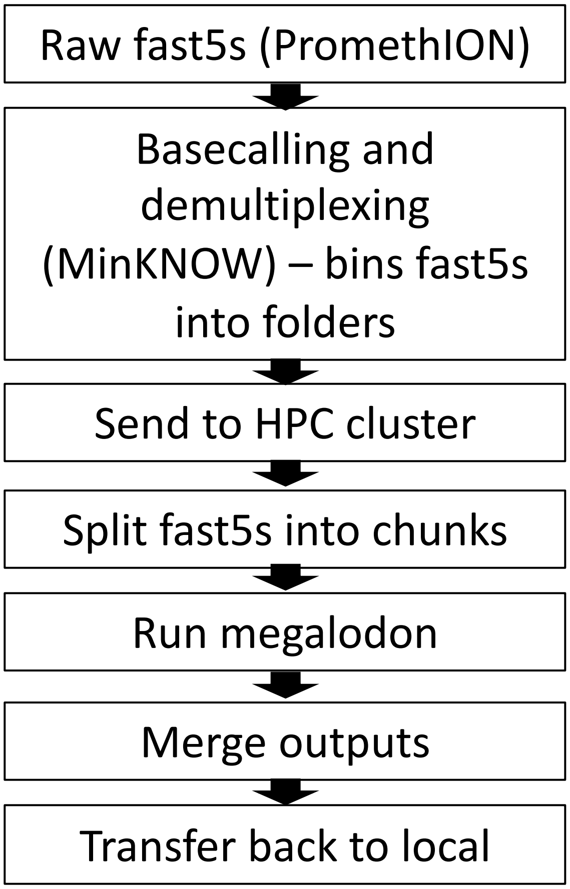

Supplementary Figure 3. Computational workflow. The workflow for calling methylation from nanopore-based cfDNA sequencing data is shown. These steps enable streamlined processing of large data volumes (>10TB).

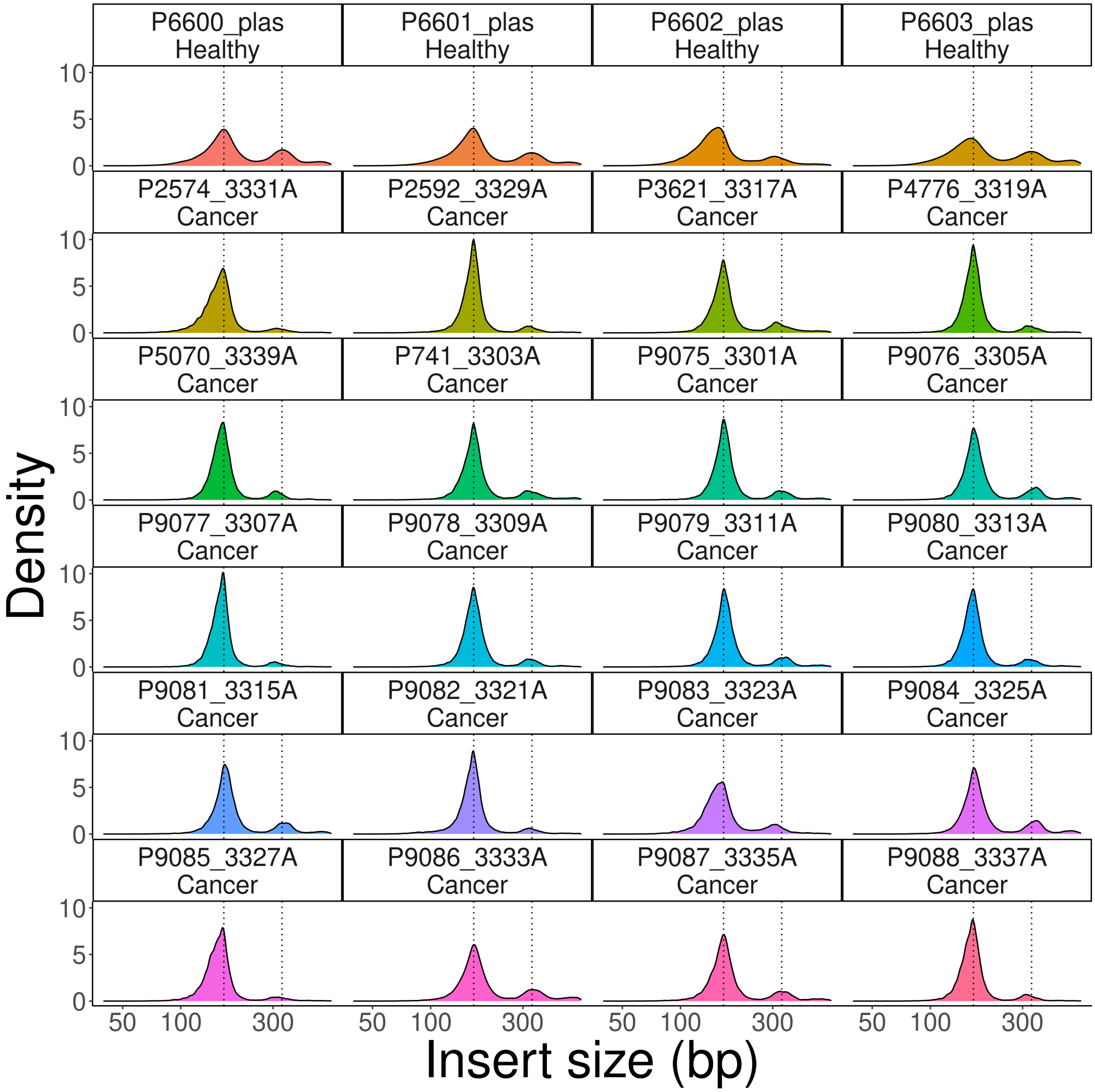

Supplementary Figure 4. Fragment size distribution of healthy donor and cancer patient cfDNA. The fragment size distribution of healthy control plasma and cancer patient-derived plasma is shown. The top row consists of cfDNA samples from healthy controls. The remaining rows come from cancer patients. Dotted lines indicated mono- and di-nucleosomes.

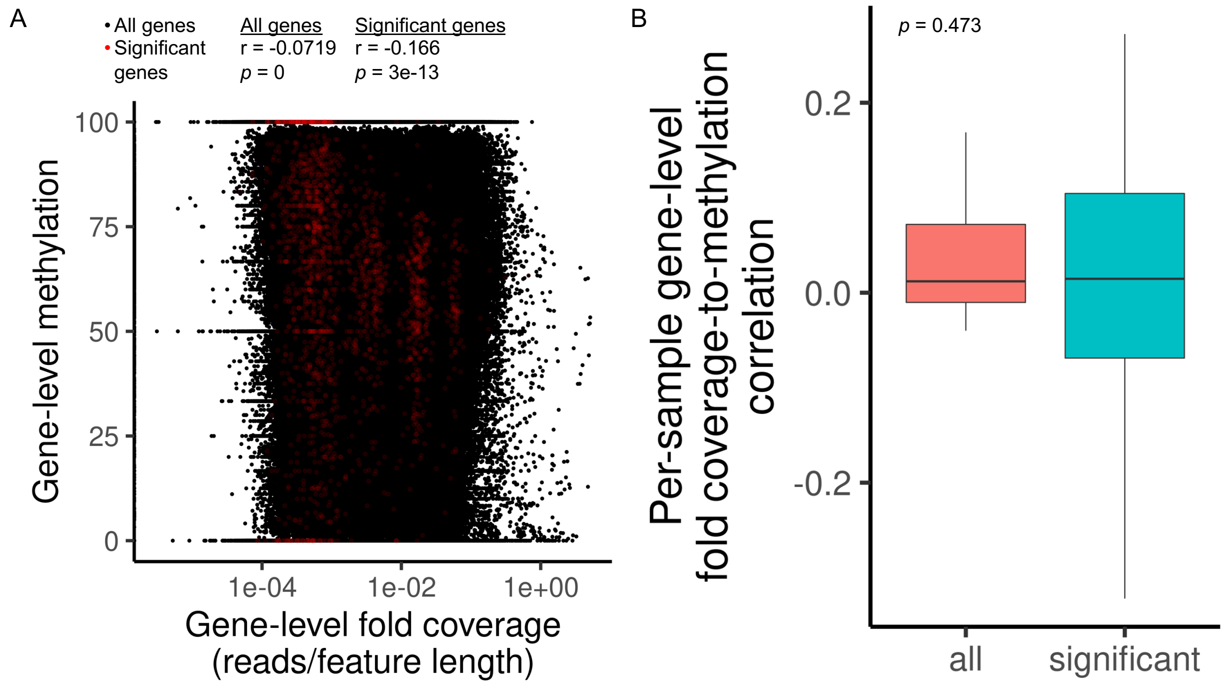

Supplementary Figure 5. Correlation between gene-level fold coverage and gene-level methylation. (A) The fold coverage of a specific genomic feature and its corresponding methylation is plotted. Fold coverage is defined as number of reads divided by the length of the feature. A single point represents a particular gene for one sample. Black: all features are considered. Red: genes found to be statistically significant between cancer patients and healthy controls. The overall Pearson correlation coefficient for all features and statistically shown genes is also displayed. (B) Per-sample fold-coverage to methylation correlation. The Pearson correlation of the gene-level methylation versus the gene-level fold-coverage is shown for each individual sample. A t-test was performed between the correlation when calculated on all genes versus only statistically significant genes, demonstrating that the differences in their correlation were not statistically significant. This shows that the statistically significant genes were selecting for differences in sequencing coverage.

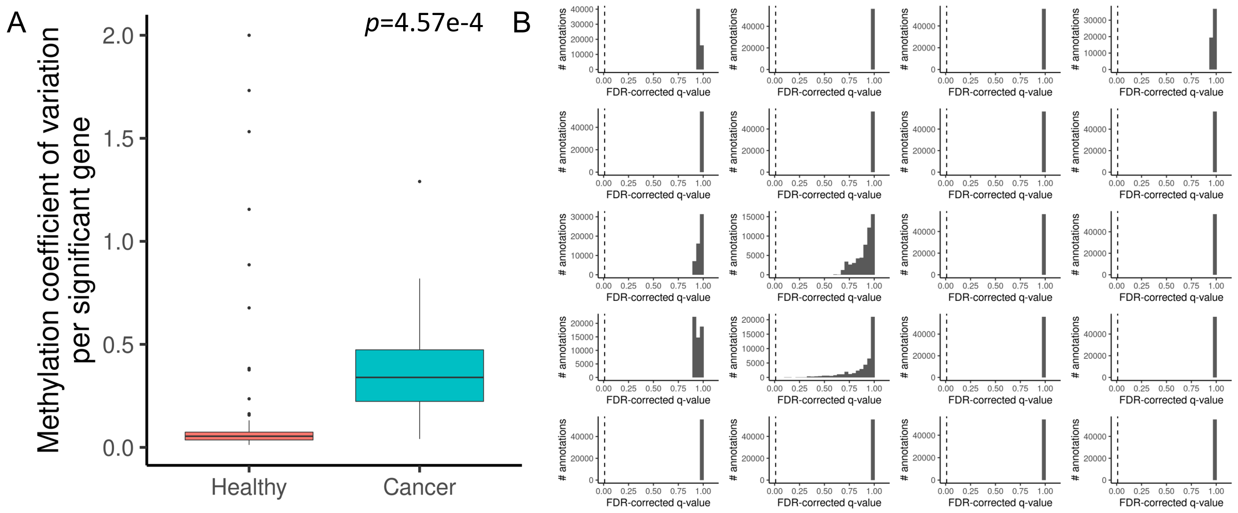

Supplementary Figure 6. Analysis of variability in gene-level methylation. (A) Analysis of within-group variability for differentially methylated genes. We identified genes with different levels of methylation when comparing cfDNA from cancer patient versus healthy controls. Genes that passed an FDR-based multiple-testing significance value of $q < 0.01$ were considered to have differential methylation. We calculated the coefficient of variation for the methylation values of each gene based on cancer patients versus controls. **(B) Random grouping.** We randomly assigned the healthy controls and cancer patients into random groups and attempted to discover differentially methylated genes. After FDR-multiple testing correction, there were zero gene annotations passing the $q < 0.01$ threshold. We repeated this process 20 times. Each facet represents the q-value distribution for each trial. The dashed line represents the 0.01 threshold.

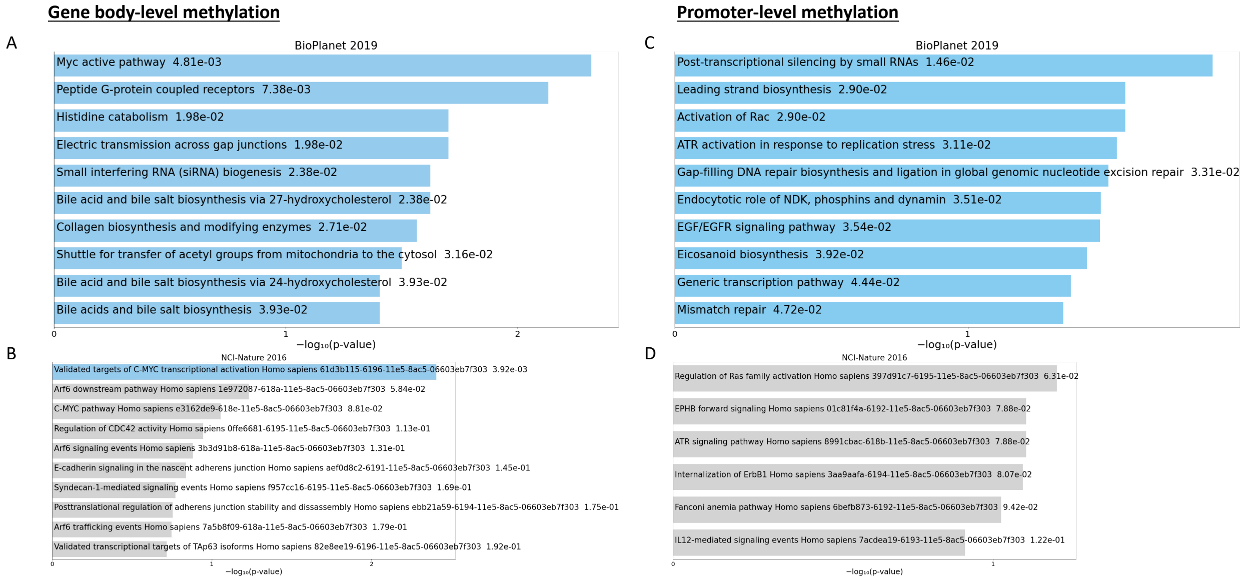

Supplementary Figure 7. Enrichment analysis for cancer patient cohort. Gene-level (left) and promoter-level (right) enrichment analysis was performed for significantly different genes between healthy and cancer patient-derived cfDNA. p-values are shown, alongside the associated pathway. Blue bars indicate $p<0.05$. A, C and B, D refer to two separate gene pathway sets curated by EnrichR.

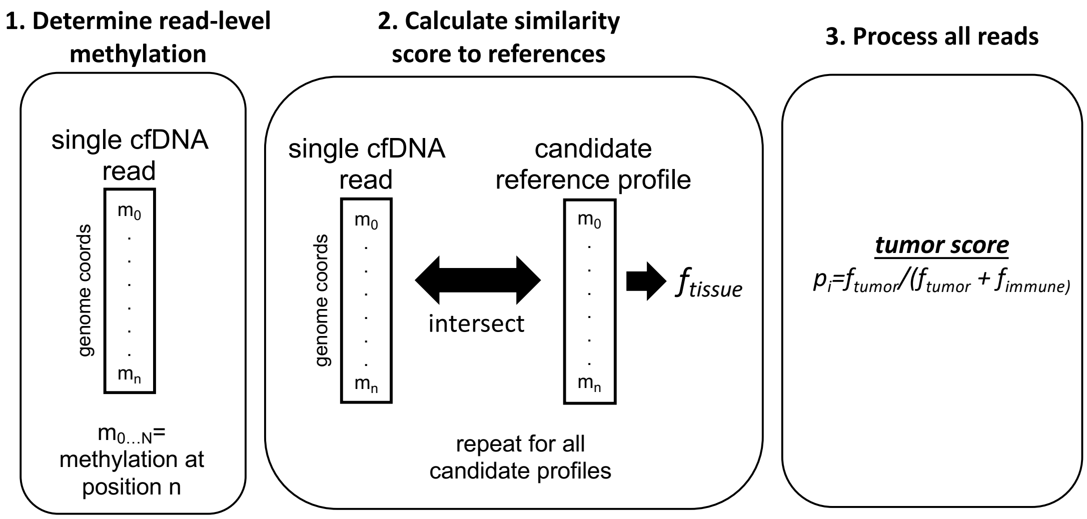

Supplementary Figure 8. Framework for read classification. Individual nanopore reads from cfDNA are classified by using reference profiles that come from matched tumor or PBMC/immune cell methylation data. Each read, their associated CpG sites, and their methylation states, are compared to candidate references. The calculated score reflects the similarity of a read to a particular candidate reference methylome. Regardless of their methylation status, all reads were processed with this framework.

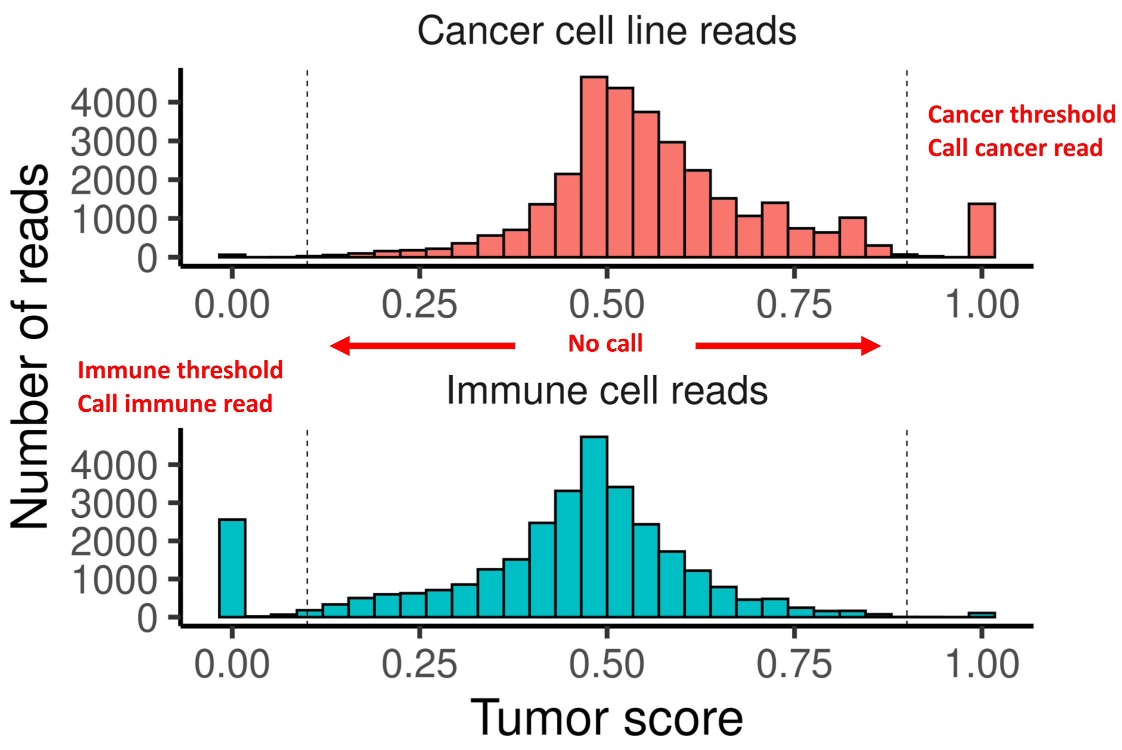

Supplementary Figure 9. Distribution of tumor classification scores for single reads. We provide an example of a tumor score histogram using an *in silico* admixture read data set. Each read has a calculated tumor score based on its methylation similarity to a matched tumor or immune reference profile. The title of each panel reflects the ground truth origin of each read set. Cancer reads are sequences that are mixed from GP2D cancer cell line nucleosomes that were nanopore sequenced and for which methylation calls were made, and immune cell reads are reads that are from healthy donor nucleosomes. There are two classification thresholds: one for immune cell origin, and one for classification of cancer cell origin. Reads matching the threshold criteria, such as tumor score > 0.9 or < 0.1, are classified as tumor-specific or immune-specific respectively. Reads falling outside the thresholds are not classified and are excluded from analysis.

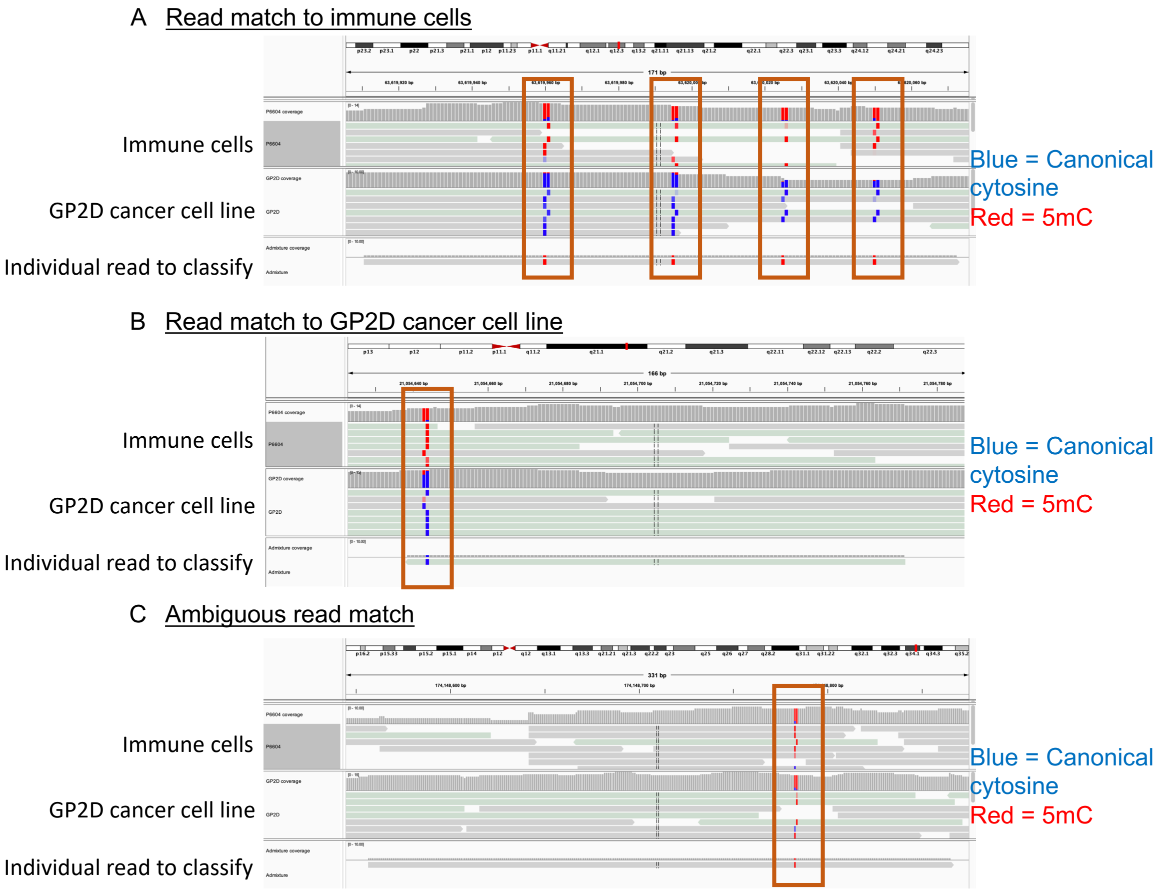

Supplementary Figure 10. Examples of read classification. The methylation profiles of immune cells and the GP2D cancer cell line are shown in selected regions, along with an individual read to classify. The shaded bases correspond to a CpG site; blue represents a canonical cytosine, while red corresponds to a detected 5mC. An *in silico* mixture of reads from both sources are sampled. An individual read is classified if its methylation matches (A) immune cells, or (B) the cancer cell line GP2D. Ambiguous matches (eg. regions where the methylation is the similar for both samples) are shown in (C).

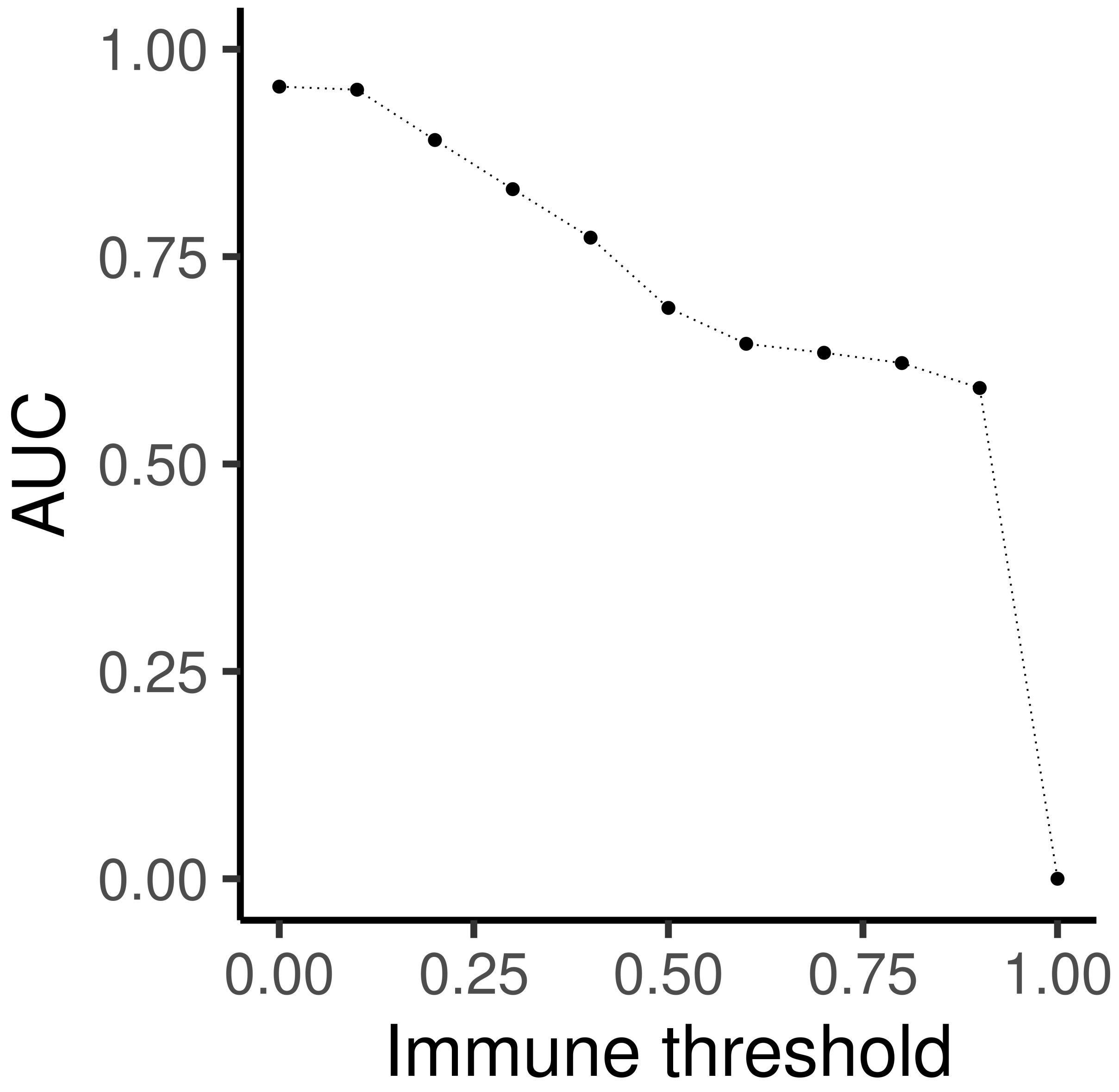

Supplementary Figure 11. Classification AUC for various thresholds. The AUC is calculated for various immune threshold values for one set of an *in silico* admixture between cancer cell line and healthy donor methylome data.

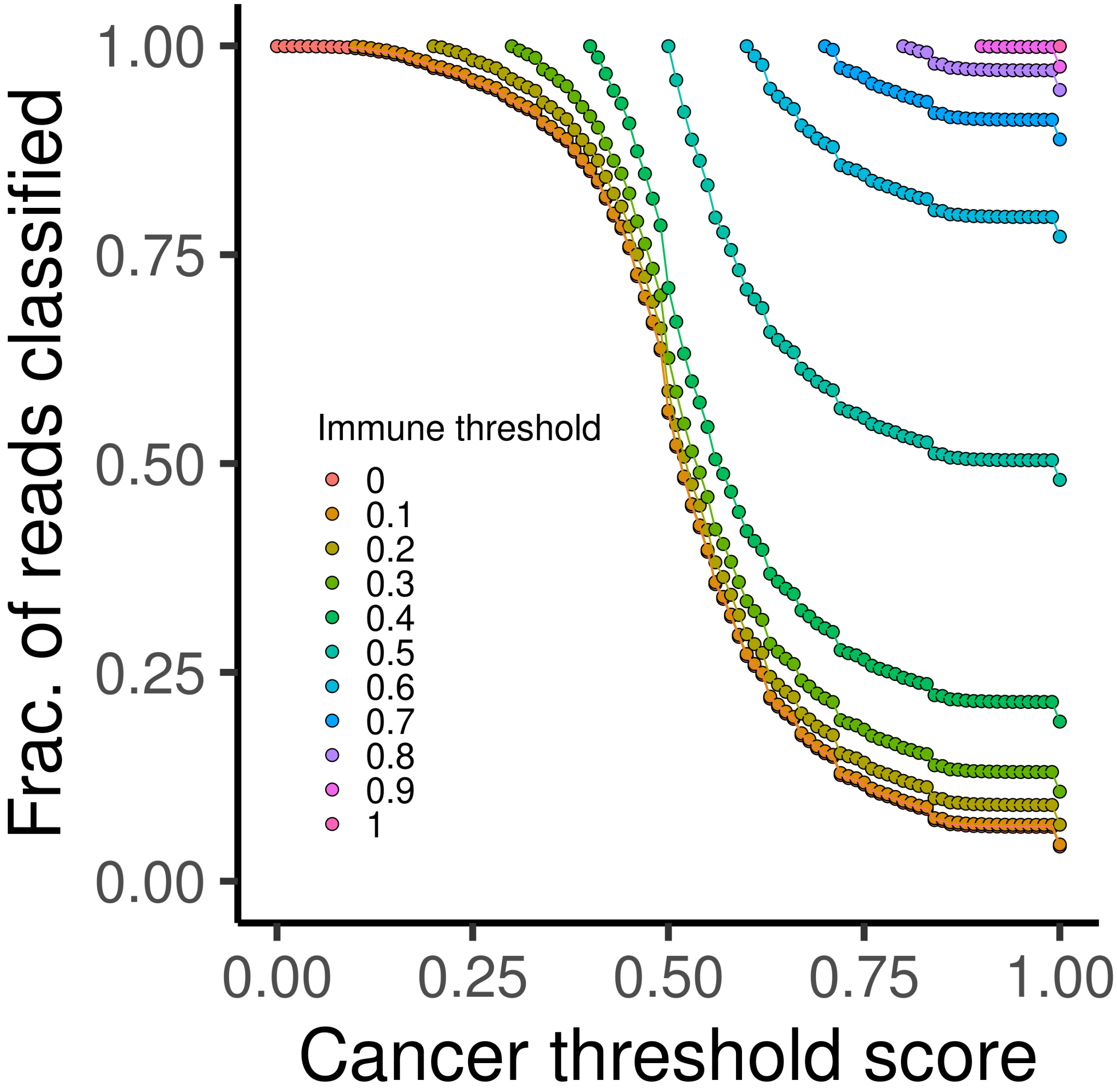

Supplementary Figure 12. Fraction of reads classified. The proportion of reads being classified is shown for various immune threshold values for one set of an *in silico* mixture between cancer cell line and healthy donor methylome data.

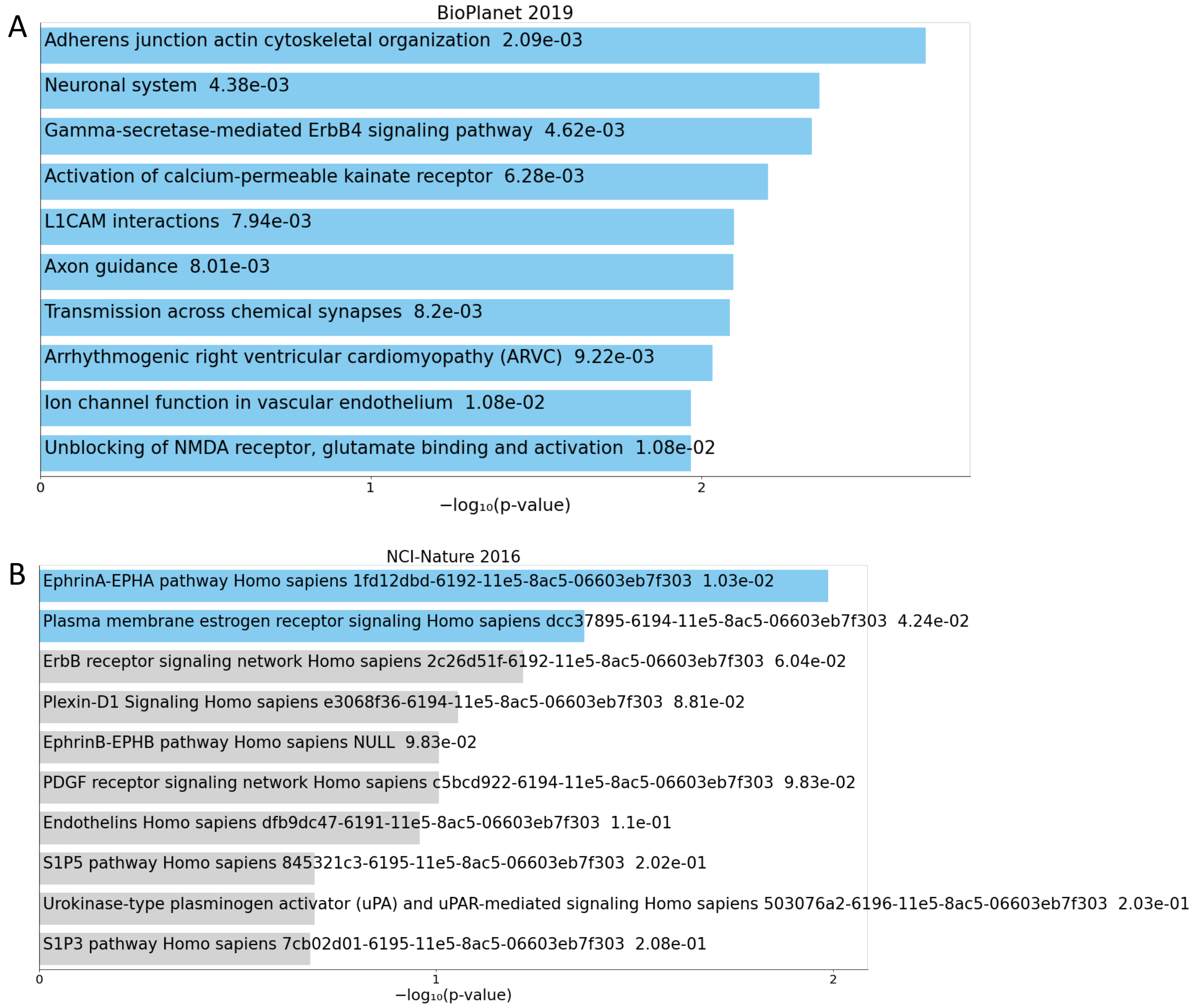

Supplementary Figure 13. Gene enrichment analysis for single molecular classifier using a GP2D cancer cell line and immune cell model. Regions with maximum methylation differences (eg. either 100% methylated in GP2D and 0% methylated in immune cells, or vice versa) between the cancer cell line and immune cells are intersected with GENCODE v38 gene-level annotations. These features are then subject to pathway enrichment analysis using different curated pathway sets in EnrichR.

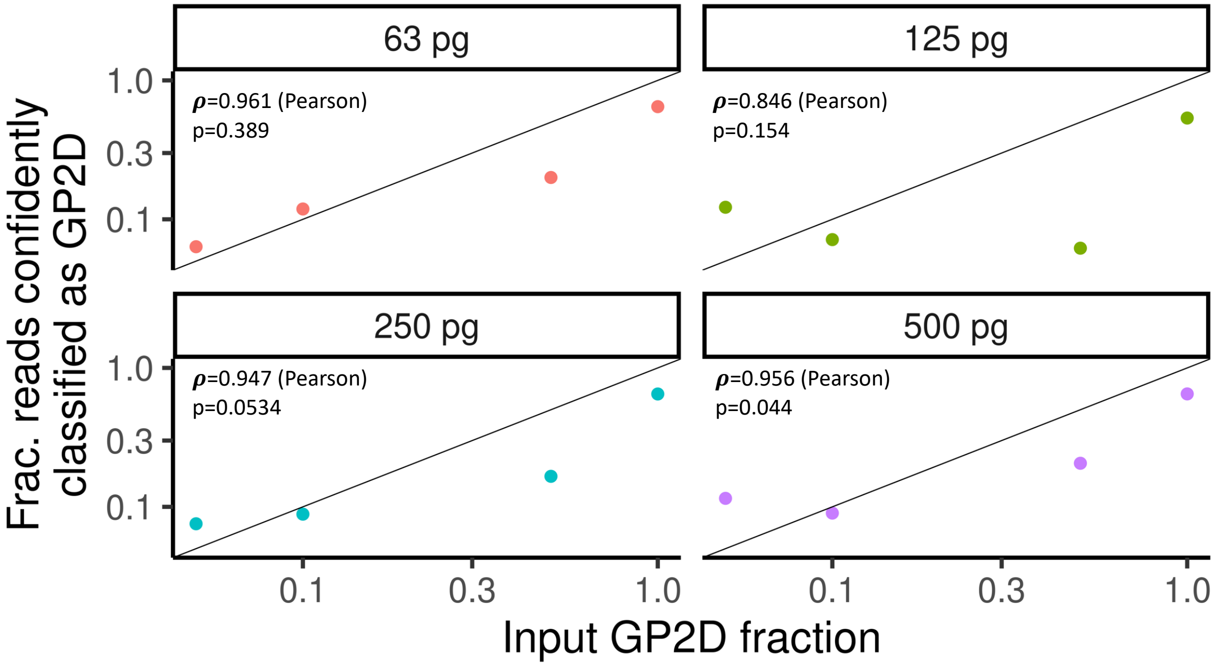

Supplementary Figure 14. Experimental admixtures. Experimental admixtures were performed between digested nucleosomes of the cancer cell line GP2D and healthy donor PBMCs. Various mixture fractions and input amounts are shown.

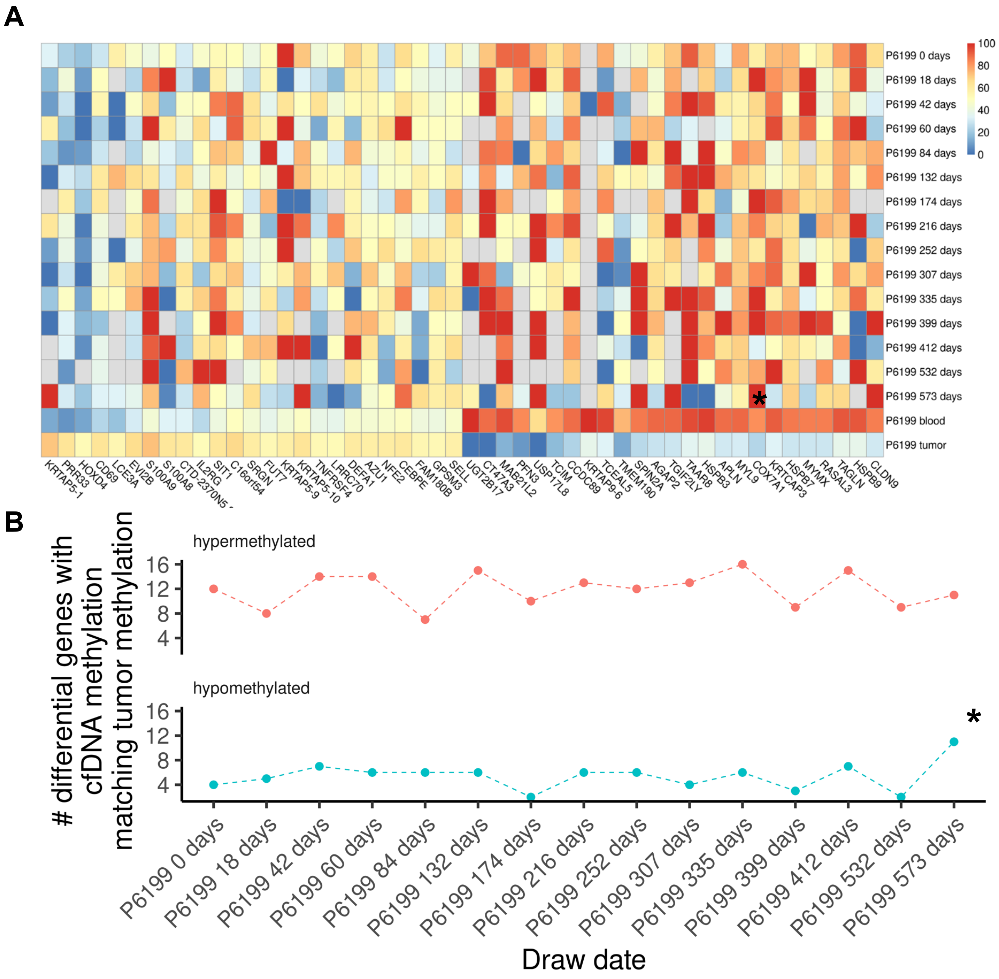

Supplementary Figure 15. Gene-level visualization for longitudinally collected plasma samples for patient P6199. (A) Gene-level methylation is shown from the analysis of longitudinal cfDNA data as well as the matched tumor and immune methylomes. The top and bottom 25 genes with differing methylation between the primary tumor and immune cells were selected. Gray boxes indicate no reads were obtained for that sample. (B) The number of tumor-specific differentially methylated genes found to be matching in cfDNA is shown for each time point. Differentially methylated genes were defined as those with the largest difference in methylation between the primary tumor and immune cells. Such methylated genes observed in cfDNA are defined as matching the primary tumor when its methylation state (eg. hypermethylation or hypomethylation) is concordant. Specific time points are annotated with asterisks to denote clinical events with significant changes in methylation.

P4822 – Metastatic pancreatic neuroendocrine carcinoma

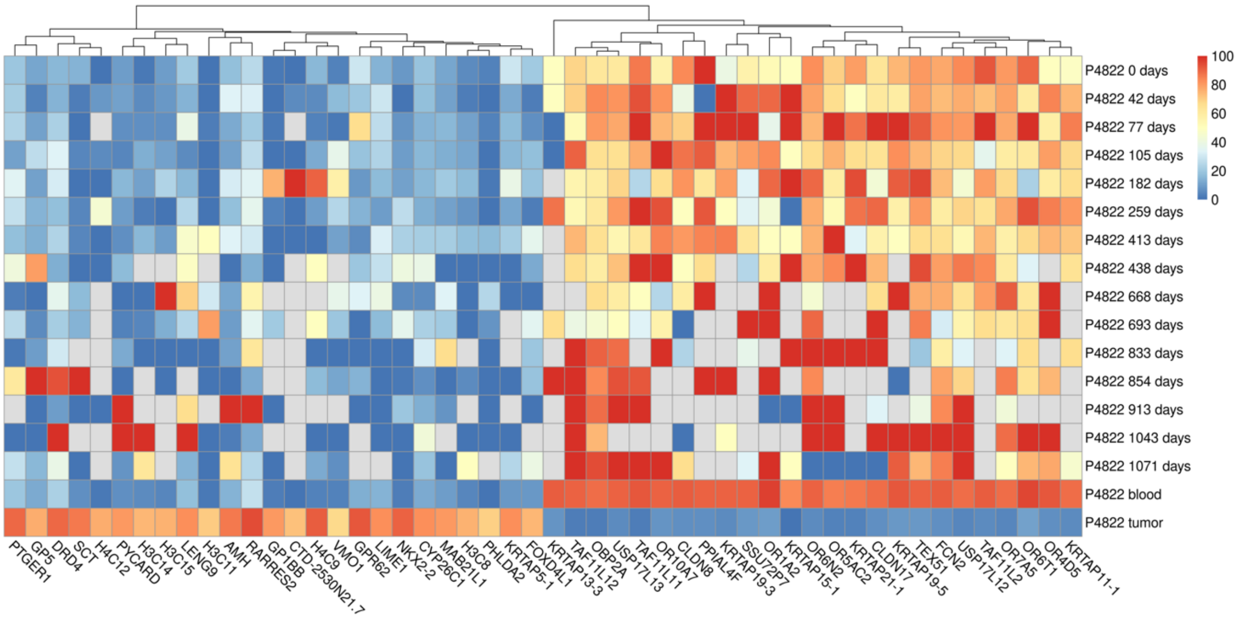

Supplementary Figure 16. Gene-level visualization for patient P4822 with metastatic pancreatic neuroendocrine carcinoma. Gene-level methylation is shown from the analysis of longitudinal cfDNA data as well as the matched tumor and immune methylomes. The top and bottom 25 genes with differing methylation between the primary tumor and immune cells were selected. Gray boxes indicate no reads were obtained for that sample.

P6527 – Intrahepatic cholangiocarcinoma

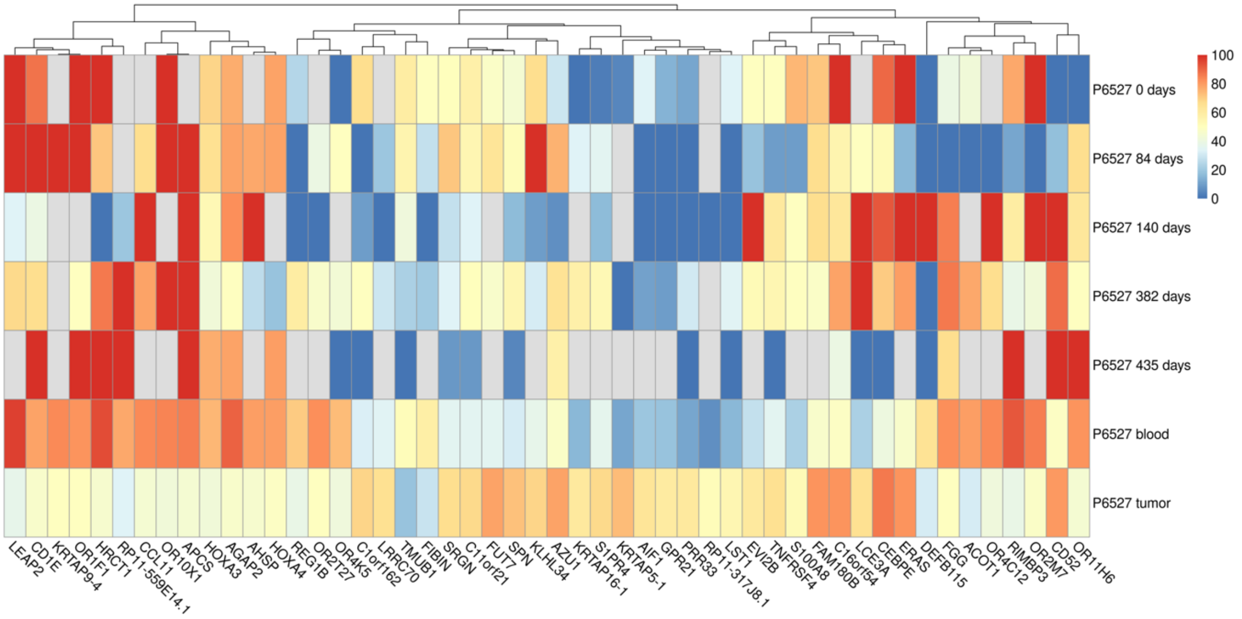

Supplementary Figure 17. Gene-level visualization for patient P6527 with metastatic cholangiocarcinoma. Gene-level methylation is shown from the analysis of longitudinal cfDNA data as well as the matched tumor and immune methylomes. The top and bottom 25 genes with differing methylation between the primary tumor and immune cells were selected. Gray boxes indicate no reads were obtained for that sample.

Supplementary Table 1. Sequencing Metrics

Patient	Sample	Experiment/Cohort	# reads	# aligned	% aligned	# CpG sites sequenced	Sequencing run batch ID	Run N50	Run Gbases > Q7	Number of flow cells
P6604	P6604_nucl	Protocol Comparison - This work - 5ng - replicate 1	7.34M	6.47M	88%	N/A	1234	285	17.05	1
P6604	P6604_nucl	Protocol Comparison - This work - 5ng - replicate 2	7.85M	6.73M	86%	N/A	1234	285	17.05	1
P6604	P6604_nucl	Protocol Comparison - This work - 5ng - replicate 3	9.49M	4.70M	50%	N/A	1234	285	17.05	1
P6604	P6604_nucl	Protocol Comparison - This work - 1ng - replicate 1	1.84M	1.44M	78%	N/A	1234	285	17.05	1
P6604	P6604_nucl	Protocol Comparison - This work - 1ng - replicate 2	1.61M	1.31M	82%	N/A	1234	285	17.05	1
P6604	P6604_nucl	Protocol Comparison - This work - 1ng - replicate 3	1.69M	1.36M	81%	N/A	1234	285	17.05	1
P6604	P6604_nucl	Protocol Comparison - This work - 0.5ng - replicate 1	.89M	.71M	79%	N/A	1234	285	17.05	1
P6604	P6604_nucl	Protocol Comparison - This work - 0.5ng - replicate 2	1.37M	.82M	60%	N/A	1234	285	17.05	1
P6604	P6604_nucl	Protocol Comparison - This work - 0.5ng - replicate 3	.81M	.63M	77%	N/A	1234	285	17.05	1
P6604	P6604_nucl	Protocol Comparison - This work - 0.1ng - replicate 1	.21M	.13M	60%	N/A	1234	285	17.05	1
P6604	P6604_nucl	Protocol Comparison - This work - 0.1ng - replicate 2	.37M	.16M	42%	N/A	1234	285	17.05	1
P6604	P6604_nucl	Protocol Comparison - This work - 0.1ng - replicate 3	.34M	.14M	43%	N/A	1232	260	1.78	1
P6604	P6604_nucl	Protocol Comparison - ONT NBD196 protocol - 5ng - replicate 1	1.57M	1.34M	85%	N/A	1232	260	1.78	1
P6604	P6604_nucl	Protocol Comparison - ONT NBD196 protocol - 5ng - replicate 2	1.49M	1.26M	84%	N/A	1232	260	1.78	1
P6604	P6604_nucl	Protocol Comparison - ONT NBD196 protocol - 5ng - replicate 3	1.43M	1.22M	85%	N/A	1232	260	1.78	1
P6604	P6604_nucl	Protocol Comparison - ONT NBD196 protocol - 1ng - replicate 1	.33M	.27M	84%	N/A	1232	260	1.78	1
P6604	P6604_nucl	Protocol Comparison - ONT NBD196 protocol - 1ng - replicate 2	.34M	.28M	84%	N/A	1232	260	1.78	1
P6604	P6604_nucl	Protocol Comparison - ONT NBD196 protocol - 1ng - replicate 3	.43M	.36M	84%	N/A	1232	260	1.78	1
P6604	P6604_nucl	Protocol Comparison - ONT NBD196 protocol - 0.5ng - replicate 1	.12M	.10M	83%	N/A	1232	260	1.78	1
P6604	P6604_nucl	Protocol Comparison - ONT NBD196 protocol - 0.5ng - replicate 2	.13M	.10M	83%	N/A	1232	260	1.78	1
P6604	P6604_nucl	Protocol Comparison - ONT NBD196 protocol - 0.5ng - replicate 3	.13M	.11M	84%	N/A	1232	260	1.78	1
P6604	P6604_nucl	Protocol Comparison - ONT NBD196 protocol - 0.1ng - replicate 1	.03M	.02M	82%	N/A	1232	260	1.78	1
P6604	P6604_nucl	Protocol Comparison - ONT NBD196 protocol - 0.1ng - replicate 2	.03M	.03M	80%	N/A	1232	260	1.78	1
P6604	P6604_nucl	Protocol Comparison - ONT NBD196 protocol - 0.1ng - replicate 3	.03M	.02M	81%	N/A	1232	260	1.78	1
GP2D	GP2D_nucl	In silico admixture	97.06M	90.64M	93%	56.41M	1195	548	41.97	1
P6604	P6604_nucl	In silico admixture	115.41M	101.09M	88%	42.62M	1196	281	28.79	1
Admixture	Admixture	Experimental admixture - 500 pg - 100% GP2D	.41M	.11M	26%	.15M	1172	296	2.78	1
Admixture	Admixture	Experimental admixture - 500 pg - 50% GP2D	.64M	.18M	29%	.16M	1172	296	2.78	1
Admixture	Admixture	Experimental admixture - 500 pg - 10% GP2D	.51M	.23M	44%	.17M	1172	296	2.78	1
Admixture	Admixture	Experimental admixture - 500 pg - 5% GP2D	.66M	.27M	40%	.19M	1172	296	2.78	1
Admixture	Admixture	Experimental admixture - 250 pg - 100% GP2D	.33M	.05M	16%	.08M	1172	296	2.78	1
Admixture	Admixture	Experimental admixture - 250 pg - 50% GP2D	.28M	.08M	29%	.08M	1172	296	2.78	1
Admixture	Admixture	Experimental admixture - 250 pg - 10% GP2D	.44M	.12M	26%	.08M	1172	296	2.78	1
Admixture	Admixture	Experimental admixture - 250 pg - 5% GP2D	.42M	.12M	29%	.09M	1172	296	2.78	1
Admixture	Admixture	Experimental admixture - 125 pg - 100% GP2D	.46M	.03M	7%	.04M	1172	296	2.78	1
Admixture	Admixture	Experimental admixture - 125 pg - 50% GP2D	.34M	.04M	13%	.04M	1172	296	2.78	1
Admixture	Admixture	Experimental admixture - 125 pg - 10% GP2D	.48M	.06M	12%	.04M	1172	296	2.78	1
Admixture	Admixture	Experimental admixture - 125 pg - 5% GP2D	.48M	.06M	13%	.05M	1172	296	2.78	1
Admixture	Admixture	Experimental admixture - 63 pg - 100% GP2D	.27M	.02M	8%	.03M	1172	296	2.78	1
Admixture	Admixture	Experimental admixture - 63 pg - 50% GP2D	.41M	.04M	10%	.04M	1172	296	2.78	1
Admixture	Admixture	Experimental admixture - 63 pg - 10% GP2D	.45M	.05M	11%	.04M	1172	296	2.78	1
Admixture	Admixture	Experimental admixture - 63 pg - 5% GP2D	.15M	.05M	32%	.04M	1172	296	2.78	1
P6600	P6600_plas	20 patient cohort - Healthy Control	3.01M	1.37M	46%	3.02M	1140	360	50.22	2
P6601	P6601_plas	20 patient cohort - Healthy Control	4.56M	1.69M	37%	3.71M	1140	360	50.22	2
P6602	P6602_plas	20 patient cohort - Healthy Control	6.97M	2.10M	30%	3.12M	1140	360	50.22	2
P6603	P6603_plas	20 patient cohort - Healthy Control	2.64M	.66M	25%	1.77M	1140	360	50.22	2
P2574	P2574_3331A	20 patient cohort - Cancer	16.42M	14.54M	89%	11.66M	1051	270	173.67	4
P2592	P2592_3329A	20 patient cohort - Cancer	2.19M	1.95M	89%	2.07M	1051	270	173.67	4
P3621	P3621_3317A	20 patient cohort - Cancer	1.89M	1.68M	89%	2.26M	1051	270	173.67	4
P4776	P4776_3319A	20 patient cohort - Cancer	3.71M	3.37M	91%	3.41M	1051	270	173.67	4
P5070	P5070_3339A	20 patient cohort - Cancer	197.66M	179.80M	91%	52.48M	1051	270	173.67	4
P741	P741_3303A	20 patient cohort - Cancer	1.67M	1.26M	76%	2.39M	1051	270	173.67	4
P9075	P9075_3301A	20 patient cohort - Cancer	1.60M	1.40M	88%	1.69M	1051	270	173.67	4
P9076	P9076_3305A	20 patient cohort - Cancer	2.59M	2.37M	91%	2.94M	1051	270	173.67	4
P9077	P9077_3307A	20 patient cohort - Cancer	72.23M	64.74M	90%	33.69M	1051	270	173.67	4
P9078	P9078_3309A	20 patient cohort - Cancer	59.52M	54.30M	91%	35.20M	1051	270	173.67	4
P9079	P9079_3311A	20 patient cohort - Cancer	2.01M	1.71M	85%	2.11M	1051	270	173.67	4
P9080	P9080_3313A	20 patient cohort - Cancer	10.37M	9.45M	91%	8.83M	1051	270	173.67	4
P9081	P9081_3315A	20 patient cohort - Cancer	2.11M	1.63M	77%	2.14M	1051	270	173.67	4
P9082	P9082_3321A	20 patient cohort - Cancer	13.85M	12.40M	90%	10.34M	1051	270	173.67	4
P9083	P9083_3323A	20 patient cohort - Cancer	52.56M	47.41M	90%	30.97M	1051	270	173.67	4
P9084	P9084_3325A	20 patient cohort - Cancer	2.82M	2.47M	88%	3.35M	1051	270	173.67	4
P9085	P9085_3327A	20 patient cohort - Cancer	8.91M	7.88M	88%	6.78M	1051	270	173.67	4
P9086	P9086_3333A	20 patient cohort - Cancer	.81M	.67M	83%	2.64M	1051	270	173.67	4
P9087	P9087_3335A	20 patient cohort - Cancer	1.35M	1.20M	89%	2.11M	1051	270	173.67	4
P9088	P9088_3337A	20 patient cohort - Cancer	54.10M	49.21M	91%	29.96M	1051	270	173.67	4
P6199	P6199_19520	Longitudinal	25.65M	22.92M	89%	17.18M	1205	267	183.5	5
P6199	P6199_19526	Longitudinal	11.40M	10.20M	90%	7.72M	1205	267	183.5	5
P6199	P6199_19572	Longitudinal	17.67M	15.86M	90%	13.55M	1205	267	183.5	5
P6199	P6199_19585	Longitudinal	9.10M	8.08M	89%	6.25M	1205	267	183.5	5
P6199	P6199_19620	Longitudinal	9.89M	8.93M	90%	8.85M	1205	267	183.5	5
P6199	P6199_21306	Longitudinal	27.27M	24.86M	91%	18.69M	1205	267	183.5	5
P6199	P6199_21347	Longitudinal	6.03M	5.46M	91%	6.46M	1205	267	183.5	5
P6199	P6199_21381	Longitudinal	13.33M	12.02M	90%	12.43M	1205	267	183.5	5
P6199	P6199_21433	Longitudinal	11.91M	10.67M	90%	9.70M	1205	267	183.5	5
P6199	P6199_21465	Longitudinal	9.46M	8.47M	90%	8.28M	1205	267	183.5	5
P6199	P6199_21488	Longitudinal	10.23M	9.32M	91%	9.63M	1205	267	183.5	5
P6199	P6199_21536	Longitudinal	4.87M	4.18M	86%	4.51M	1036	343	119.13	4
P6199	P6199_21549	Longitudinal	6.02M	5.41M	90%	6.44M	1036	343	119.13	4
P6199	P6199_21641	Longitudinal	3.13M	2.67M	85%	2.88M	1036	343	119.13	4
P6199	P6199_21671	Longitudinal	7.70M	6.87M	89%	7.08M	1036	343	119.13	4
P6199	P6199_immune	Longitudinal	14.94M	14.65M	98%	56.86M	1146	2.61k	41.91	1
P6199	P6199_primary	Longitudinal	29.43M	29.04M	99%	57.99M	1147	2.51k	50.44	1
P4822	P4822_18277	Longitudinal	57.12M	51.23M	90%	30.81M	1205	267	183.5	5
P4822	P4822_18284	Longitudinal	23.61M	20.94M	89%	17.07M	1205	267	183.5	5
P4822	P4822_18309	Longitudinal	26.81M	24.14M	90%	19.17M	1205	267	183.5	5
P4822	P4822_18317	Longitudinal	46.14M	41.39M	90%	27.94M	1205	267	183.5	5
P4822	P4822_18404	Longitudinal	33.10M	29.69M	90%	22.19M	1205	267	183.5	5
P4822	P4822_18452	Longitudinal	37.91M	33.91M	89%	24.78M	1205	267	183.5	5
P4822	P4822_19469	Longitudinal	30.17M	26.79M	89%	18.89M	1205	267	183.5	5
P4822	P4822_19502	Longitudinal	12.90M	11.51M	89%	8.03M	1205	267	183.5	5
P4822	P4822_21383	Longitudinal	8.46M	7.60M	90%	6.03M	1205	267	183.5	5
P4822	P4822_21431	Longitudinal	8.28M	7.24M	87%	7.13M	1205	267	183.5	5
P4822	P4822_21518	Longitudinal	4.35M	3.87M	89%	3.97M	1205	267	183.5	5
P4822	P4822_21540	Longitudinal	4.64M	4.02M	87%	4.21M	1205	267	183.5	5
P4822	P4822_21581	Longitudinal	2.52M	2.26M	90%	2.05M	1205	267	183.5	5
P4822	P4822_21682	Longitudinal	2.55M	2.24M	88%	2.50M	1205	267	183.5	5
P4822	P4822_22186	Longitudinal	4.93M	4.44M	90%	5.38M	1205	267	183.5	5
P4822	P4822_immune	Longitudinal	13.08M	12.90M	99%	57.90M	1223	17.82k	81.89	1
P4822	P4822_primary	Longitudinal	27.39M	26.97M	98%	57.70M	1206	8.18k	80.27	1
P6527	P6527_19633	Longitudinal	4.71M	3.82M	81%	5.29M	1139	367	91.96	4
P6527	P6527_21349	Longitudinal	6.43M	5.05M	79%	6.83M	1139	367	91.96	4
P6527	P6527_21416	Longitudinal	4.45M	3.31M	74%	3.75M	1139	367	91.96	4
P6527	P6527_21602	Longitudinal	16.68M	14.96M	90%	18.36M	1139	367	91.96	4
P6527	P6527_21639	Longitudinal	2.78M	1.90M	68%	2.38M	1139	367	91.96	4
P6527	P6527_immune	Longitudinal	17.30M	16.99M	98%	58.23M	1150	6.09k	96.39	1
P6527	P6527_primary	Longitudinal	31.53M	31.07M	99%	58.27M	1151	5.82k	90.1	1

Supplementary Table 2. Patient Information

Patient	Sequencing run batch ID	TNM staging reported at surgery	Number of time points	Primary tumor available/sequenced
P4822	Metastatic Pancreatic Neuroendocrine Carcinoma	N/A	14	Y
P6199	Invasive Adenocarcinoma, Poorly Differentiated, Extending Into Pericolonic Soft Tissue	ypT3 pN2b	15	Y
P6527	Intrahepatic Cholangiocarcinoma, Moderately Differentiated	ypT2 pN0	5	Y
P9075	Invasive Adenocarcinoma	pT3 pN2a	1	N
P741	Moderately Differentiated Colorectal Adenocarcinoma	pT4 pN2 pM1	1	N
P9076	Recurrent Adenocarcinoma, Colorectal Primary	pT3 pN1a	1	N
P9077	Invasive Adenocarcinoma	pT3 pN1b pM1a	1	N
P9078	Invasive Adenocarcinoma	ypT2 ypN0 ypM0	1	N
P9079	Adenocarcinoma, Cribriform Comedo Type	pT2 pN0	1	N
P9080	Invasive Adenocarcinoma/Metastatic Adenocarcinoma	ypT2 ypN2a ypM1	1	N
P9081	Invasive Adenocarcinoma	ypT4b ypN0	1	N
P3621	Invasive Colorectal Adenocarcinoma	pT3 pN0	1	N
P4776	Invasive Adenocarcinoma	pT4b pN1b	1	N
P9082	Metastatic Adenocarcinoma, Colorectal Primary	pT4b pN1b	1	N
P9083	Metastatic Adenocarcinoma	N/A	1	N
P9084	Metastatic Adenocarcinoma, Colorectal Primary	N/A	1	N
P9085	Metastatic Adenocarcinoma, Colorectal Primary	ypT4b pN0 pM1a	1	N
P2592	Metastatic Adenocarcinoma In Three Of Four Lymph Nodes	N/A	1	N
P2574	Metastatic Adenocarcinoma, Colorectal Primary	T3N0	1	N
P9086	Metastatic Adenocarcinoma,Colorectal Primary	ypT1 pN1c pM1a	1	N
P9087	Metastatic Adenocarcinoma, Colonic Primary	pT3 pN1a	1	N
P9088	Metastatic Adenocarcinoma, Colorectal Primary	N/A	1	N
P5070	Metastatic Adenocarcinoma	N/A	1	N

Supplementary Table 3. Genes with significant methylation differences between healthy and patient-derived cfDNA in 20 patient cohort

Gene	q-value (fdr adjusted)	Mean difference between groups (healthy - cancer)
SPIB	3.70E-06	-41.39933674
CDCA7	6.52E-06	-53.22244908
TMEM164	2.36E-05	18.216444
COL10A1	0.00015059	17.13642991
PLSCR4	0.000182298	-34.87794316
SLC25A1	0.000182298	60.89159323
ELAC2	0.000447691	25.31557705
ZNF572	0.000503628	-59.14379304
ENPP4	0.000600976	45.02995548
GPER1	0.000616948	26.90489456
PLAGL2	0.000616948	37.94148816
RPUSD4	0.000616948	37.03039294
NUF2	0.000836028	-20.19624653
SMIM10L2A	0.00083887	54.84387916
GJC1	0.001031567	33.99002109
ILRUN	0.001163916	10.93166634
ZNF414	0.001198392	-60.93005356
ZNF772	0.001198392	35.83525929
ELL2	0.00154547	-30.51454594
KLHL11	0.00154547	33.69456263
LGR4	0.00160918	-20.65861339
LHCGR	0.001730388	20.15967146
ADGRG4	0.002000412	29.8121374
CTD-2545M3.6	0.002000412	-41.10365785
ELAPOR2	0.002000412	17.26097167
MGMT	0.002000412	9.027538374
NME1	0.002177991	-51.33222625
NRTN	0.002240827	20.54819725
OSGEPL1	0.002240827	33.21504636
AMDHD1	0.002413003	36.1677824
MCHR2	0.002413003	25.94704782
ROGDI	0.002413003	43.62026154
ZNF774	0.002664044	33.96611201
RER1	0.003040081	26.67522645
OPN1MW2	0.00313277	29.30895359
TUG1	0.00313277	44.06717689
CAPN11	0.003149901	24.94768262
MRPL52	0.003149901	-40.1475143
KIAA1143	0.003187013	24.34567563
INTS6L	0.00397628	27.17036478
ARMCX5-GPRASP2	0.004016938	29.95589663
PSMD2	0.004016938	35.58255254
PUS3	0.004285724	43.49319279
LAMP2	0.004563378	22.05689646
TBC1D22A	0.004563378	5.681992433
TRIM51	0.004563378	43.78787467
UCN	0.004563378	73.24983018
CD79A	0.005618985	-48.92757848
DNHD1	0.005909211	11.01400976
ADAMTS14	0.006008943	12.90419821
RNA5S5	0.006008943	51.83139542
ULBP3	0.006008943	33.96751199
EPPIN	0.006158961	44.53709195
SSU72	0.006158961	20.63218936
ZBTB22	0.006220455	-38.11904119
FAIM	0.006354143	34.27565271
TCTA	0.006503272	52.32468329
OR4D2	0.006583676	39.60481249
RETSAT	0.006583676	32.24024626
RNFT1	0.006583676	-46.78586976
HSD3B7	0.006606003	36.44872641
NXF2	0.006695395	17.65769516
DICER1	0.007310389	14.5453317
CNKSR2	0.007376806	22.62391933
MAGEA9B	0.007376806	32.9689437
GSTT2B	0.007446381	42.19318471
TCL1B	0.007446381	30.32851384
KAT2A	0.007576995	35.28736586
NFE2L3	0.007683316	22.96929258
TMEM150A	0.008110172	50.78549013
ZIC3	0.008110172	-14.66498911
LAMTOR4	0.008516659	34.7124756
CSDE1	0.008686958	-23.89282634
MRPS18A	0.008952295	27.97364516
CCDC71	0.009340928	43.7421799
CCL16	0.009555689	33.05517035
LAMTOR2	0.009555689	48.04111602
OR13A1	0.009555689	34.48973445
GPATCH4	0.009717914	-44.38019697
EPS8L3	0.009912777	15.53967346

Supplementary Table 4. Promoter regions with significant methylation differences between healthy and patient-derived cfDNA in 20 patient cohort

Gene	q-value (fdr adjusted)	Mean difference between groups (healthy - cancer)
ADAMTS5	0.000744332	74.3651579
ATP6V1C2	0.000744332	55.08625782
GOLGA3	0.001040288	-65.84314568
LMBR1L	0.001143591	-52.98385531
ZNF70	0.001740186	68.29381334
IQCE	0.001868279	43.21712284
MCTS2P	0.001868279	-73.84181981
NARS1	0.001868279	52.76182828
PRMT6	0.001868279	-62.78849577
RBM3	0.001868279	-62.67878454
RFC4	0.001868279	-67.19968029
TNRC6A	0.001868279	57.38201821
ZNF302	0.001868279	62.41968858
ZNF347	0.001868279	66.00328427
ZNF547	0.00226444	-40.24916908
LTA4H	0.003035531	-66.28461522
AC003002.4	0.003871603	-39.316108
GRAMD2B	0.003871603	-67.33218618
TRAPPC2B	0.003871603	-39.316108
TRIM37	0.004194124	63.26893732
NCKIPSD	0.004398915	-56.20384937
ABRAXAS2	0.004432868	50.25232108
TMC6	0.004432868	-45.85008315
TSSK4	0.004432868	-74.37285432
S100A6	0.004875013	63.98208544
TASOR	0.005014826	54.74321695
LACTB2	0.005026056	51.95163346
NUMBL	0.005026056	-46.12731636
SYNJ1	0.005026056	44.01968376
HLX	0.005736971	-26.0744204
C11orf49	0.006799178	49.14447271
SOS2	0.006799178	47.26719909
TRA2B	0.006865967	53.18400708
CCDC63	0.007253305	40.34577853
TATDN3	0.007253305	53.71590157
TRNT1	0.007253305	60.64754018
ZNF512	0.007253305	43.96701187
RP11-529K1.3	0.007464227	-50.32722662
MRPL53	0.008000474	55.96806399
ZNRF2	0.009027935	58.57515035
TCAP	0.009356029	44.62004723
ANKRD52	0.009626255	57.78896067

Supplementary Table 5. Methods Comparison

Method	Sequencing Platform	Resolution	PCR-free	Input requirement	Comments
This work	Oxford Nanopore	Base-pair	Yes	pg to ng	Utlizes LSK110 latest chemistry on R9.4.1 flow cells
Conventional Nanopore	Oxford Nanopore	Base-pair	Yes	>40ng	Barcoding adapters are stuck with an previous generation sequencing adapter
Bisulfite	llumina	Base-pair	No	tens to hundreds of ng	
Enzymatic	llumina	Base-pair	No	tens to hundreds of ng	
cfMeDIP-Seq	Illumina	Binned	Yes	ng to hundreds of ng	requires carrier
